# Exploration of Gas-Dependent Self-Adaptive Reconstruction Behavior of Cu_2_O for Electrochemical CO_2_ Conversion to Multi-Carbon Products

**DOI:** 10.1007/s40820-024-01568-1

**Published:** 2024-11-19

**Authors:** Chaoran Zhang, Yichuan Gu, Qu Jiang, Ziyang Sheng, Ruohan Feng, Sihong Wang, Haoyue Zhang, Qianqing Xu, Zijian Yuan, Fang Song

**Affiliations:** State Key Laboratory of Metal Matrix Composites, School of Materials Science and Engineering, Shanghai Jiao Tong University, Shanghai, 200240 People’s Republic of China

**Keywords:** CO_2_ reduction reaction, Electrocatalysts, Cu_2_O, Reconstruction, Self-adaptive electrocatalysis

## Abstract

Structural reconstruction of electrocatalysts plays a pivotal role in catalytic performances for CO_2_ reduction reaction (CO_2_RR), whereas the behavior is by far superficially understood. Here, we report that CO_2_ accessibility results in a universal self-adaptive structural reconstruction from Cu_2_O to Cu@Cu_x_O composites, ending with feeding gas-dependent microstructures and catalytic performances. The CO_2_-rich atmosphere favors reconstruction for CO_2_RR, whereas the CO_2_-deficient one prefers that for hydrogen evolution reaction. With the assistance of spectroscopic analysis and theoretical calculations, we uncover a CO_2_-induced passivation behavior by identifying a reduction-resistant but catalytic active Cu(I)-rich amorphous layer stabilized by *CO intermediates. Additionally, we find extra CO production is indispensable for the robust production of C_2_H_4_. An inverse correlation between durability and FE_CO_/FE_C2H4_ is disclosed, suggesting that the self-stabilization process involving the absorption of *CO intermediates on Cu(I) sites is essential for durable electrolysis. Guided by this insight, we design hollow Cu_2_O nanospheres for durable and selective CO_2_RR electrolysis in producing C_2_H_4_. Our work recognizes the previously overlooked passivation reconstruction and self-stabilizing behavior and highlights the critical role of the local atmosphere in modulating reconstruction and catalytic processes.

**Supplementary Information:**

The online version contains supplementary material available at 10.1007/s40820-024-01568-1.

## Highlights

- We revealed a universal self-adaptive structural reconstruction from Cu_2_O to Cu@Cu_x_O composites, ending with feeding gas-dependent microstructures and catalytic performances.
- We uncovered a CO_2_-induced passivation behavior by identifying a reduction-resistant but catalytic active Cu(I)-rich amorphous layer.
- We designed and fabricated hollow Cu_2_O nanospheres, demonstrating durable electrolysis at a partial current density of −200 mA cm^−2^ in producing C_2_H_4_ with an FE of up to 61% at −0.6 V_RHE_.

**Supplementary Information:**

The online version contains supplementary material available at 10.1007/s40820-024-01568-1.

## Graphical abstract

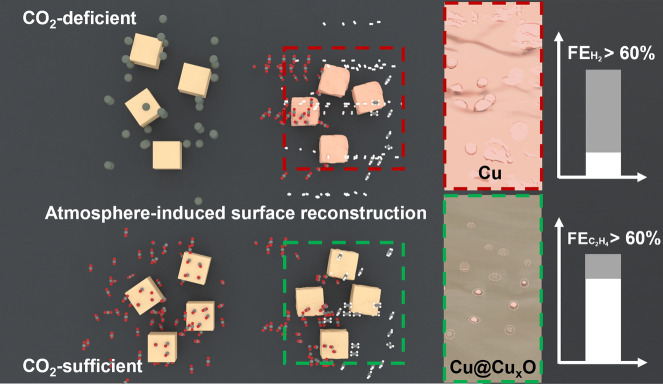

## Introduction

Electrocatalytic CO_2_ reduction to chemical feedstocks and value-added products coupled with intermittent renewable energy is a promising approach to warrant carbon neutrality [1, 2]. Electrocatalysts play a pivotal role in energy-effective conversion. Among them, Cu-based materials have attracted the most research interest, as they can reduce CO_2_ to a wide range of hydrocarbons and oxygenates (especially the C_2+_ products) [3–6]. Strategies including selective facet exposure [7], morphology design [8], electronic state modulation [9, 10], surface modification [11], valence modulation/oxide reconstruction [12], etc. have been utilized to optimize the catalytic performances. However, it remains challenging to prompt the C_2+_ products selectively and effectively, due to the sluggish CO_2_ activation and coupling of carbon–carbon bonds (C–C) and the deactivation of catalysts [10, 13, 14].

In comparison with polycrystalline bulk-like Cu [15–17], their oxides and derivatives (Cu_*x*_O) favor value-added multi-carbon products [18–20]. Cu(I) species are suggested to be of vital significance in this process [21–23], due to the favorable *CO adsorption and C–C coupling [17, 24, 25]. Unfortunately, Cu(I) species are thermodynamically unstable. It undergoes over-reduction to metallic Cu(0) (0.52 V vs. RHE), leading to the decay of activity and selectivity [26]. The transition has been ubiquitously disclosed in copper oxides (Cu_2_O or CuO) and even metallic Cu(0), accompanied by structural reconstruction in most cases [27, 28]. Much effect has been devoted to elucidating the reconstruction process, aiming to stabilize Cu(I) by interfacing, doping, pre-oxidation, or reviving them by pulse electro-reduction technique [29–31]. Due to its dynamic nature, the behavior has yet been superficially understood, leading to controversial structure-performance relationships. The performances (activity, selectivity, and durability) vary from group to group for the same catalyst [32–34]. The active phases were recognized with different compositions and microstructures, even if catalysts were fabricated by the same method. In some cases, Cu(0) was only detected, questioning the attribution of high C_2_ selectivity to the presence of Cu(I) for C–C coupling [35].

Besides catalysts themselves, microenvironments including gas accessibility, local pH, and triple-phase interface are equally important [36, 37]. The impact of microenvironments on practical electrolyzers has been well investigated but with much emphasis on linking them to catalytic processes. It should be noted that redox/chemical reactions are usually coupled with reconstruction and occur prior to or concurrently with catalytic reactions. In this sense, microenvironments could modulate redox/chemical reactions and the related reconstruction behavior in a similar way as catalytic reactions. However, this has yet been largely overlooked. Wang et al. showed that the CO_2_ atmosphere preferred electrodeposition of Cu-based catalyst favorable for C_2_H_4_ [38]. A specific *CO binding was suggested to be vital in guiding the preferential facet growth for good selectivity. Alivisatos et al. revealed that feeding gases could drive diverse reconstruction of metallic Cu nanocrystals under catalysis [39]. Ren et al. observed nanoscale surface roughness on the Cu surface induced by a strong affinity of CO under CO_2_-sufficient conditions [40]. Niu et al. demonstrated that the CO_2_ atmosphere facilitates the Lanthanum leaching, thereby exposing active Cu species in orthorhombic-type perovskite La_2_CuO_4_ pre-catalyst [41]. These preliminary results indicate a strong link between atmosphere and structural reconstruction. However, how they interact with each other and further with the catalytic process is still vague.

**Scheme 1:**
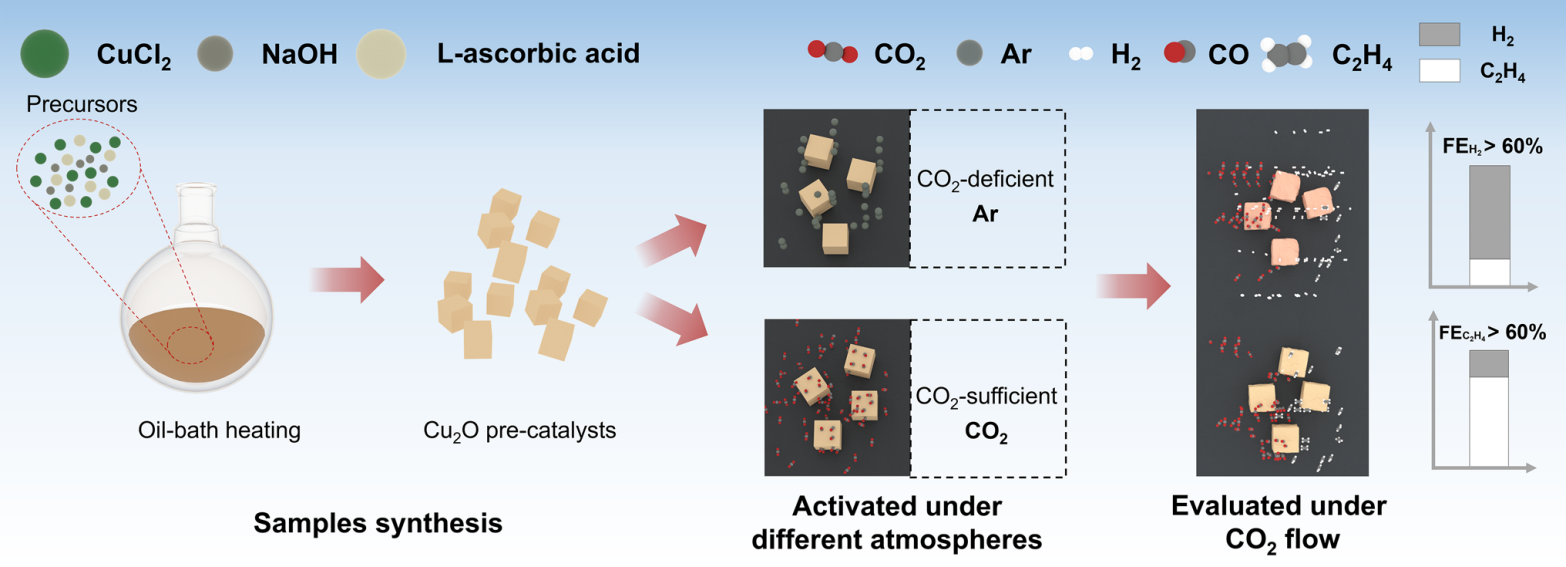
Illustration of sample synthesis, CO_2_ accessibility-dependent reconstruction, and catalytic performances

Here, we investigate the influence of feeding gas on the structural reconstruction of CO_2_RR catalysts using well-defined Cu(I)-rich Cu_2_O as pre-catalysts and further link it to catalytic activity and durability. Though the catalytic process and structural evolution of Cu_2_O have been well investigated [40, 42, 43], their dependence on feeding gas has been unexplored to the best of our knowledge. Revisiting the reconstruction by taking gas accessibility into account could provide additional information to rationalize previous debates [44, 45]. Cu_2_O is electrochemically activated in the Ar atmosphere to monitor the reconstruction in CO_2_-deficient regions and compared to that activated in the CO_2_ atmosphere (Scheme 1). Microstructural and spectroscopic analysis is then conducted to identify the structural reconstruction and link it to catalytic processes and performances. A CO_2_-induced passivation behavior is disclosed in the reconstruction process. Furthermore, a self-stabilization behavior involving the absorption of *CO intermediates is uncovered in the catalytic process. The strong affinity between metastable Cu(I) sites and *CO intermediates is highlighted and suggested to be essential in structural reconstruction, C–C bond coupling reaction, and durable electrocatalysis. Finally, hollow Cu_2_O nanospheres favoring CO_2_-rich reconstruction are fabricated purposely, which validates our new understanding of atmosphere driving self-adaptive reconstruction and catalytic process.

## Experimental Section

### Reagents and Materials

All reagents and materials were used directly with no further purification. Copper sulfate pentahydrate (CuSO_4_·5H_2_O, 99.9%), Cupric chloride (CuCl_2_·2H_2_O, 99.99%), sodium hydroxide (NaOH, 98%–100.5%), potassium hydroxide (KOH, 99.99%), potassium bicarbonate (KHCO_3_, 99.99%), L-Ascorbic acid (C_6_H_8_O_6_, ACS) were purchased from Shanghai Aladdin Biochemical Technology Co., Ltd. Nafion^®^ (D520) dispersion was purchased from Dupont. Nickel foam, Sigracet 28BC carbon paper with a gas diffusion layer, and the anion exchange membranes (FAA-3-50, Fumapem, and Sustainion^®^ X37-50 Grade RT, Dioxide Material) were purchased from the Fuel Cell Store.

### Synthesis of Cu_2_O Nanocrystals

#### Cu_2_O Cube

10 mL CuCl_2_ solution (0.1 M) was added into 90 mL distilled water dropwise to form a homogenous solution. After the solution was heated to 55 °C for 30 min, 10 mL NaOH aqueous solution (2 M) was dropped slowly till the solution turned from blue to dark brown. After stirring for another 30 min, 10 mL of 0.6 M L-ascorbic acid was added. The above mixture was stirred for an extra 3 h before centrifugation. Then the as-prepared sample was rinsed with deionized water and ethanol. Finally, the powders were dried in a vacuum box at room temperature.

#### Cu_2_O Hollow Spheres

0.05 g CuSO_4_·5H_2_O was dissolved in 100 mL of 0.1 M CTAB aqueous solution. Then 0.18 g L-ascorbic acid was added. The above mixture was heated to 60 °C and maintained for 20 min. 0.2 M NaOH was added dropwisely into the above solution to form a yellow precipitate. After stirring for another 10 min, the precipitate was centrifuged, washed sequentially with deionized water and ethanol several times, and then dried at 50 °C under vacuum.

#### Gas-Mediated Reconstruction

The atmosphere-mediated reconstructed catalyst was accomplished via applying potential by bubbling various gases into the solution for a certain time, and then the pretreated catalyst was used for further electrocatalytic tests. During the pretreatment, the flow rate of gases was controlled at 10 s.c.c.m. via a mass-flow controller.

### Density Functional Theory Calculations

All density functional theory (DFT) calculations were performed using the Vienna ab initio simulation package (VASP). The generalized gradient approximation (GGA) with PBE functional was applied. The core electrons were approximated through projector augmented wave functions (PAW). We employed a kinetic cutoff energy of 500 eV for the plane-wave basis set. A smearing of 0.1 eV was added to facilitate the convergence of the wave function. Geometries were optimized until the energy was converged to 1.0 × 10^–6^ eV atom^–1^ and the force was converged to 0.05 eV Å^–1^. Initial structures of Cu and Cu_2_O were obtained from the Materials Project database based on our X-ray diffraction (XRD) and transmission electron microscopy (TEM) results. After building the slab models, a vacuum layer of 15 Å was added to avoid interactions between adjacent images. These slab models’ bottom two atomic layers were kept fixed during the simulations.

The Gibbs free energy change for each reaction step is calculated as:

$$\Delta G=\Sigma G\left(\text{products}\right)-\Sigma G\left(\text{reactants}\right)$$

where *G*(i) is the Gibbs free energy of species i. Gibbs free energy of each species was calculated as *G* = *E* + *ZPE* − *TS*, where *E* is the total energy obtained from DFT calculations, *ZPE* is the zero-point energy, and *S* is the entropy. Temperature *T* was set to be 298.15 K.

## Results and Discussion

### Feeding Gas-Dependent Microstructure Evolution of Cu_2_O Nanocubes

**Fig. 1:**
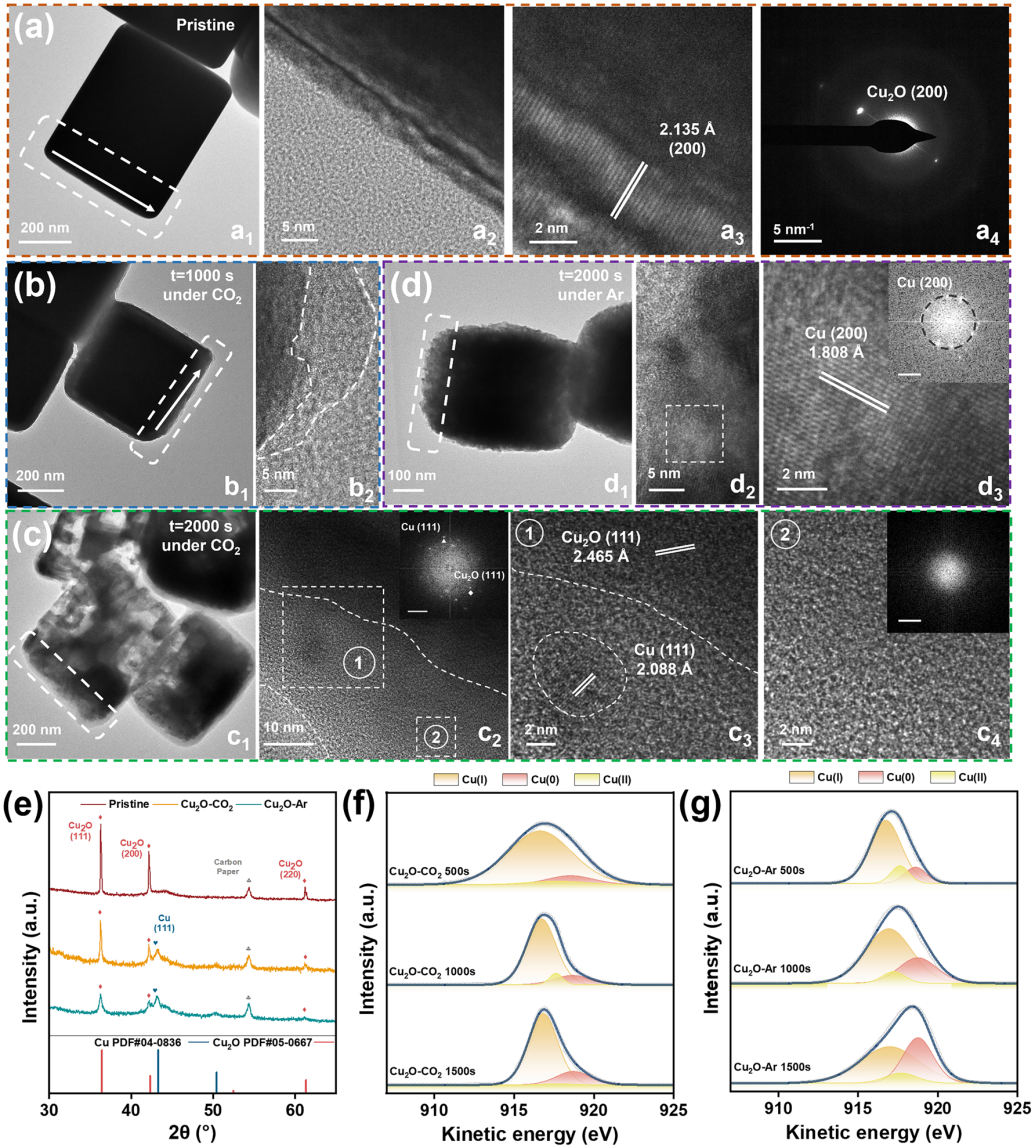
Feeding gas-dependent structural evolution of Cu_2_O nanocubes in the pre-electrolysis at a potential of −1.1 V versus RHE in an H-cell. TEM and HRTEM images and corresponding SAED patterns of **a** pristine Cu_2_O, and those after pre-electrolysis in CO_2_ atmosphere for **b** 1000 s and **c** 2000 s, and **d** after pre-electrolysis in Ar atmosphere for 2000 s; The a2 and a3 show the edge of nanocube in a1. The a4 is the SAED pattern of the nanocube in a1. The b2 shows the edge of the nanocube in b1. The d2 is the enlarged rectangle region in d1. The d3 is the enlarged square region in d2. The inset in d3 is the SAED pattern. The c3 and c4 are the enlarged square regions marked with numbers ① and ② in c2, respectively. **e** XRD patterns and **f, g** Cu LMM Auger spectra of Cu_2_O-CO_2_ and Cu_2_O-Ar after pre-electrolysis for different periods

Cu_2_O nanocubes were synthesized by a previously reported method [33] (see details in the **Experimental Section**). The scanning electron microscopy (SEM) images show that Cu_2_O nanocrystals are uniform in size of ~ 180 nm (Fig. S1). The atomic ratio of Cu and O is around 73:27 for a single cube according to the STEM EDS mapping (Fig. S1 and Table S1), indicating the presence of some O vacancy. Each nanoparticle has the nature of a single crystal, as evidenced by TEM and corresponding high-resolution transmission electron microscopy (HRTEM) images (Figs. 1a and S2). They are enclosed by (100) facets for Cu_2_O nanocubes. The clear lattice fringes in HRTEM images and the intense diffraction peaks in XRD patterns (Figs. 1a and S1) indicate the highly crystalline nature. The interplanar spacings of 2.135, 1.51, and 3.02 Å are ascribed to the (200), (220), and (110) planes of Cu_2_O nanocrystals, respectively. The XRD peaks correspond well to the cubic phase of Cu_2_O (PDF#05-0677, Figs. 1e and S1), indicating the absence of crystalline impurity.

Based on previous electrochemical analysis of Cu_2_O for CO_2_RR catalysis [33, 34], a typical potential of −1.1 V versus RHE was applied to drive the surface reconstruction for 2000 s in CO_2_ and Ar, which monitors the CO_2_-rich and CO_2_-deficient micro-environments, respectively. The samples are denoted as Cu_2_O-CO_2_ and Cu_2_O-Ar, respectively. Techniques including TEM, XRD, and Auger spectra were employed to probe the reconstruction behavior (Figs. 1 and S3–S8). In particular, the identical location TEM technique was used to trace the structural evolution at the same location (Fig. 1a–d), which gives intact information on structure change. The pristine Cu_2_O can be identified with a well-defined single-crystal structure (Figs. 1a and S3). It shows that the crystalline surface underwent amorphization in a CO_2_-rich atmosphere. The amorphous layer of Cu_*x*_O was ~ 10 nm in thickness in 1000 s’ pretreatment, and prone to expand toward the interior in prolonged electrolysis (Figs. 1b and S4). Meanwhile, the solid interior started to fragment into small nanoparticles and eventually became porous or/and hollow in 2000 s’ electrolysis (Figs. 1c and S4) [40]. Careful examination of the surface shows inner Cu_2_O nanoparticles are encapsulated by an amorphous Cu_x_O layer, where Cu(0) nanoclusters of ~ 5 nm in size are well dispersed (Figs. 1c2, c3 and S4). The elemental mapping analysis showed an atomic Cu:O ratio of 77:23 on the surface (Table S2), indicating a small amount of O atoms were extracted to form a passivating amorphous Cu_x_O layer. The SAED pattern of the core–shell structure shows diffraction patterns of both Cu(0) and Cu_2_O nanoclusters (inset in Fig. 1c2). The Cu(0) nanocluster can be distinguished by the clear lattice spacing of 2.088 Å, corresponding to (111) planes of metallic Cu. In contrast, only a rough surface was formed in the Ar atmosphere (CO_2_-deficient) (Figs. 1d and S5-S6). The thickness is over 40 nm in the 2000s pretreatment. HRTEM images and SAED rings show that the surface is almost made of metallic Cu(0) nanoclusters, with the absence of any amorphous Cu_x_O matrix. The lattice spaces of 1.808 Å correspond to the crystal plane of (200) of metallic Cu (inset in Fig. 1d3.). The elemental mapping analysis shows an Cu:O atomic ratio of 94:6 on the surface (Table S3), suggesting a high degree of transition from Cu_2_O to Cu on the surface under the Ar atmosphere. The distinct microstructures and compositions offer strong evidence of the feeding gas-dependent structure evolution and phase/composition transition.

XRD analysis over a mass of nanocrystals confirmed the partial phase transition from Cu_2_O to metallic Cu(0) in both cases (Fig. 1e). Moreover, even though the interiors of Cu_2_O were fragmented in the CO_2_ atmosphere, more Cu_2_O phase retained in Cu_2_O-CO_2_ than in Cu_2_O-Ar (as suggested by the higher peak intensity of Cu_2_O phase in the former one). This indicates the amorphous layer might act as a passivation layer to protect inner Cu_2_O nanoparticles from electrochemical reduction. Cu LMM Auger spectra were further collected from Cu_2_O-CO_2_ and Cu_2_O-Ar in the increasing period of pre-electrolysis (from 500, 1000, to 1500 s, Figs. 1f, g and S7), to trace the reducing process on surface. The spectra were deconvoluted to peaks of Cu(0), Cu(I), and Cu(II). The Cu(II) was attributed to the oxidation in air. The peak ratio of Cu(0)/Cu(I) increases with prolonged pre-electrolysis, suggesting a reducing process in both Cu_2_O-CO_2_ and Cu_2_O-Ar under the applied negative potential. Moreover, Cu_2_O-CO_2_ exhibits a much lower Cu(0)/Cu(I) ratio (0.36:1 for the 1500 s) than Cu_2_O-Ar (0.90:1 for the 1500 s), validating the high resistance of the amorphous layer to the reducing potential in Cu_2_O-CO_2_. The results are consistent with the abundant Cu(I) and a few tiny Cu nanoclusters in the amorphous layer.

**Fig. 2:**
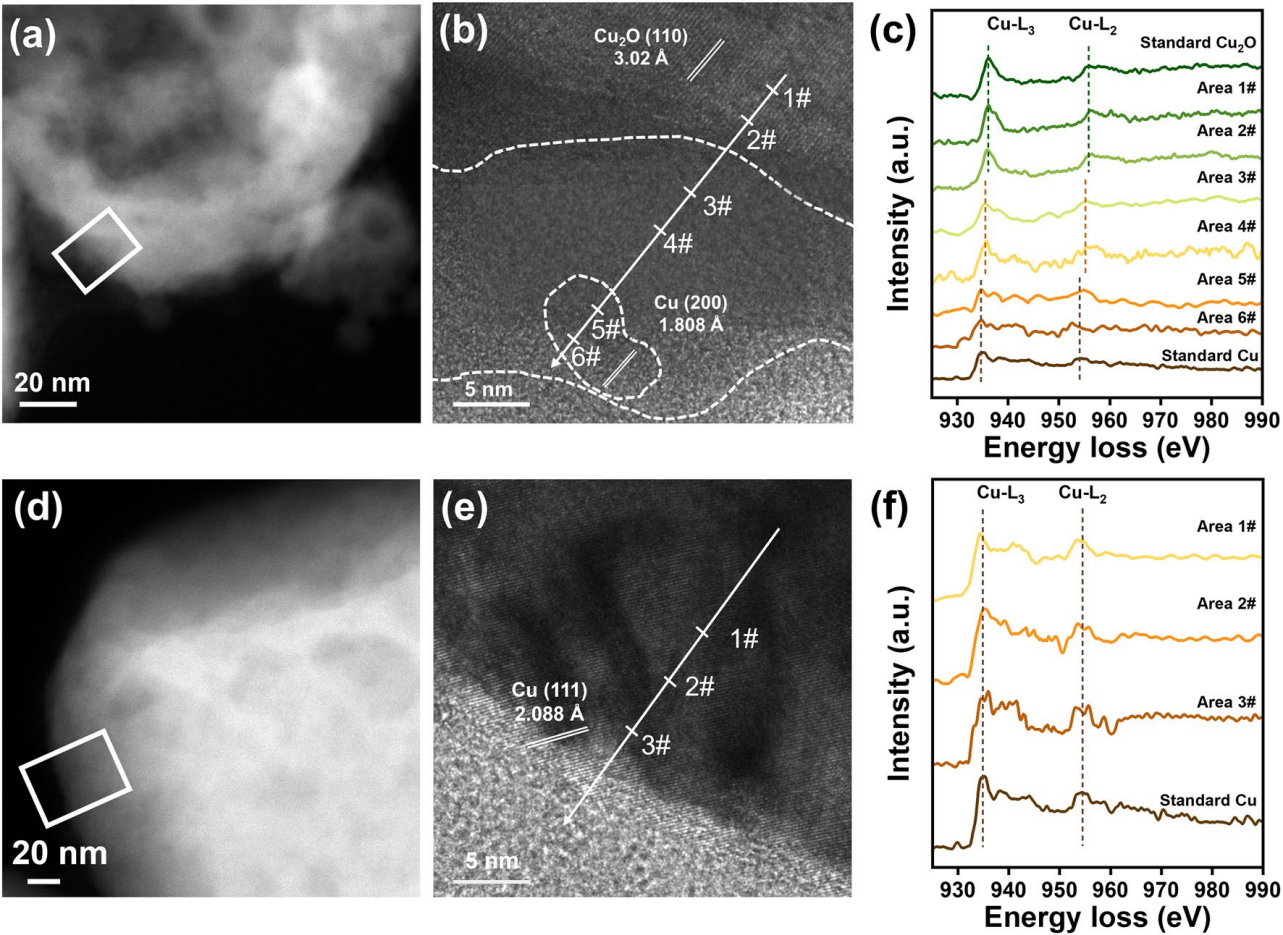
Valence state of copper on the surface of Cu_2_O-CO_2_ and Cu_2_O-Ar. **a, d** HAADF images and **b, e** HRTEM images of **a, b** Cu_2_O-CO_2_ and **d, e** Cu_2_O-Ar. The points for EELS were marked in the HRTEM images in **b** and **e**; EELS L_2,3_ edge spectra of **c** Cu_2_O-CO_2_ and **f** Cu_2_O-Ar collected from the rectangle region marked in** b** and** f**, from the inner part to the utmost surface part along the lines marked in** b** and** e**

To probe the local valence state of copper in Cu_2_O-CO_2_ and Cu_2_O-Ar, we collected the electron energy-loss spectroscopy (EELS) on the surface (Fig. 2). The spectra were recorded in the rectangle regions in Fig. 2a, d. To reveal the spatial distribution, the spectra were collected from the inner part to the utmost surface part, along the lines marked in their HETEM images in Fig. 2b, e. Region 1# and 2# in Fig. 2b were the inner crystalline Cu_2_O that was not reduced yet in Cu_2_O-CO_2_. They exhibit peaks located at 935.9 eV (Cu-L_3_) and 955.7 eV (Cu-L_2_), in line with the reference Cu_2_O sample. Region 3# and 4# were in the amorphous regions. The EELS peaks narrowed and shifted to 935.6 eV (Cu-L_3_) and 955.4 eV (Cu-L_2_). The peaks were between that of reference Cu(I) and Cu (0), indicating an in-between valence state (forming Cu_*x*_O, 0 < *x* < 1). The peak intensities were also weakened, resulting from the extraction of oxygen from Cu_2_O. Region 5# and 6# were Cu(0) clusters that were well dispersed in the amorphous layer. Their spectra exhibited wide and inconspicuous white lines, consistent with the metallic nature, where electrons were filled within the 3*d* band and no other oxygen hybridization contributed to white lines [46–49]. The peak positions at 934.5 and 954.3 eV also agree well with that of reference Cu. Differently, Cu_2_O-Ar showed only metallic Cu components (Fig. 2f), in good agreement with the results in Figs. 1 and S4–S9.

### Feeding Gas-Dependent Electrocatalytic Performances Toward CO_2_RR of Cu_2_O Nanocubes

**Fig. 3:**
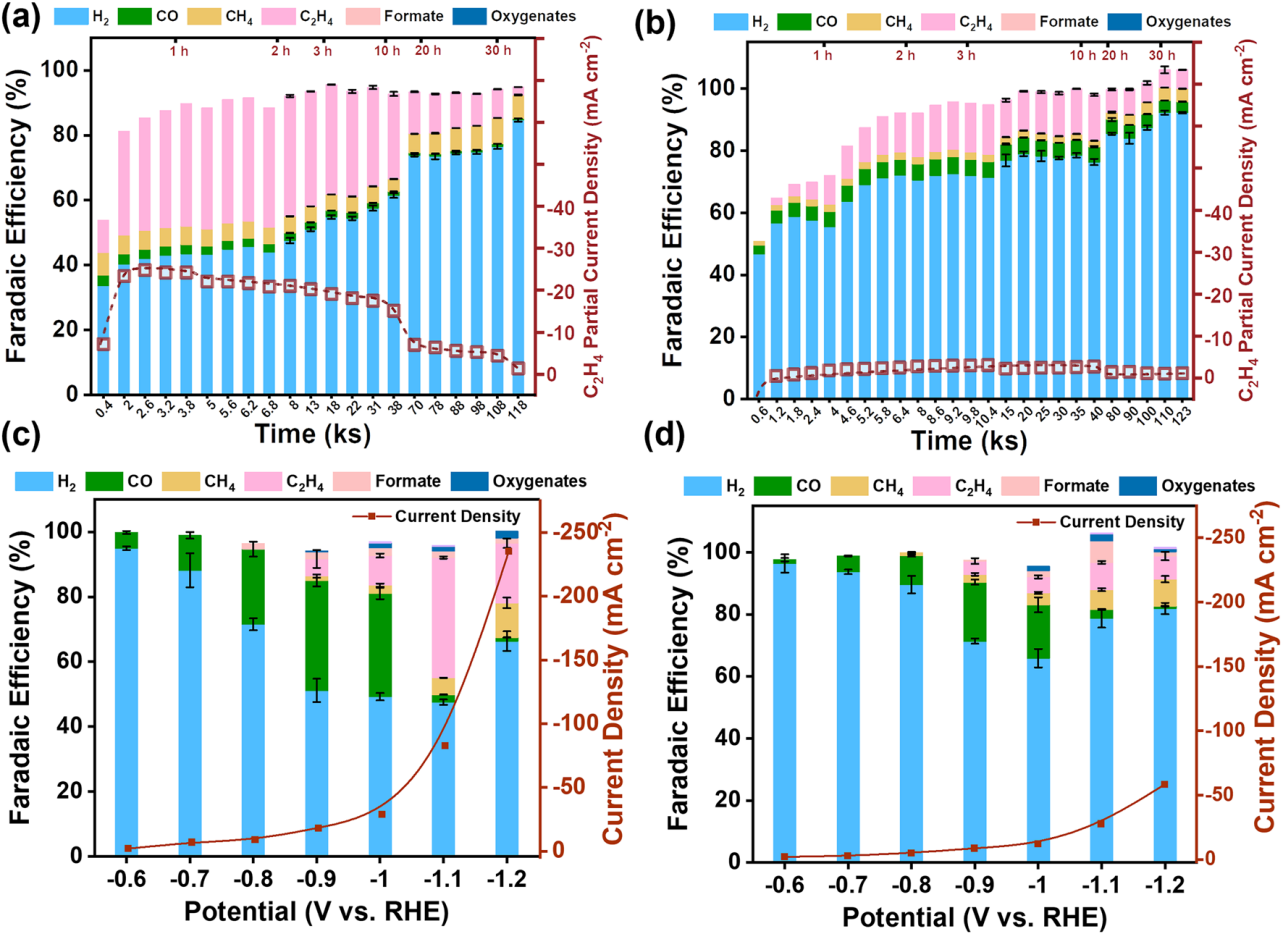
Electrochemical CO_2_RR performances of Cu_2_O nanocubes in an H-cell. Partial current density and Faradaic efficiency of the main CO_2_RR products after activation in **a** CO_2_ and **b** Ar atmosphere, respectively. The applied potential is −1.1 V versus RHE for electrolysis in CO_2_-saturated 0.5 M KHCO_3_ electrolyte; Total current density and Faradaic efficiency of the main CO_2_RR products plotting against applied potentials after activation in **c** CO_2_ and **d** Ar atmosphere, respectively. Red hollow squares are C_2_H_4_ partial current densities at each sampling time

The electrochemical CO_2_RR performances, as well as the products (Fig. S10), were then investigated to probe the catalytic response to feeding-gas-dependent reconstruction in an H-cell configuration (Fig. S11). The potential of −1.1 V versus RHE was maintained to drive CO_2_RR electrolysis for more than 30 h. The current densities were recorded, and the products were traced in specific time intervals (Fig. 3a, b**)**. After initial activation in the CO_2_ atmosphere, the main product of Cu_2_O-CO_2_ is ethylene (C_2_H_4_) (Figs. 3a and S12). The detailed gas concentration sampled at each time point is provided in Fig. S13. The partial current density reaches the peak value of −24.1 mA cm^−2^ at a faradic efficiency of 38% for C_2_H_4_, which is consistent with previously reported data (Table S4) [50, 51]. In sharp contrast, Cu_2_O-Ar can deliver a partial current density of 8 times less (−3.0 mA cm^−2^) with the faradic efficiency reduced by half (16%) (Figs. 3b and S14). The sampling information in detail is shown in Fig. S15. The higher FE for C_2_H_4_ could be attributed to the Cu(I) sites that are stabilized in the amorphous layer of Cu_2_O-CO_2_. This is in line with previous reports showing that Cu(I) sites are favorable for C–C coupling reactions [52]. Moreover, we observed that it took a long time for Cu_2_O-Ar (10,400 s) to reach the peak partial current density and Faradaic efficiency. This indicates CO_2_ would penetrate slowly into the interiors of Cu_2_O nanoparticles and drive some CO_2_-rich reconstruction for improving C_2_H_4_ selectivity in Cu_2_O-Ar. In contrast, Cu_2_O-CO_2_ reaches saturated current density and Faradaic efficiency at a rate of 5 times faster. No further CO_2_-rich reconstruction could occur in a couple of hours, likely due to the passivating effect of the amorphous layer. The current density much higher than Cu_2_O-Ar should be ascribed to the highly porous structures and the well-dispersion of the catalytic active amorphous layer through an in situ conversion process.

To obtain more information on feeding gas-dependent catalytic performances, catalysts were analyzed in a wide potential range from −0.6 to −1.2 V versus RHE (Fig. 3c, d). For both catalysts, the C_1_ compound (CO) is the dominating product below the potential of −1.0 V versus RHE, whereas C_2+_ compounds (C_2_H_4_) increase apparently above the potential. At each potential, Cu_2_O-CO_2_ exhibits better selectivity to CO_2_RR, and the current density is at least ~ 2 times higher than Cu_2_O-Ar. The FE trends for CO and C_2_H_4_ are similar in the whole potential range. Moreover, once C_2_H_4_ is produced at an appreciable rate, the CO product is much constrained. This indicates that CO intermediate could be the precursor of C_2_H_4_. Also, it might play a pivotal role in forming the passivating amorphous layer during the reconstruction process [53].

**Fig. 4:**
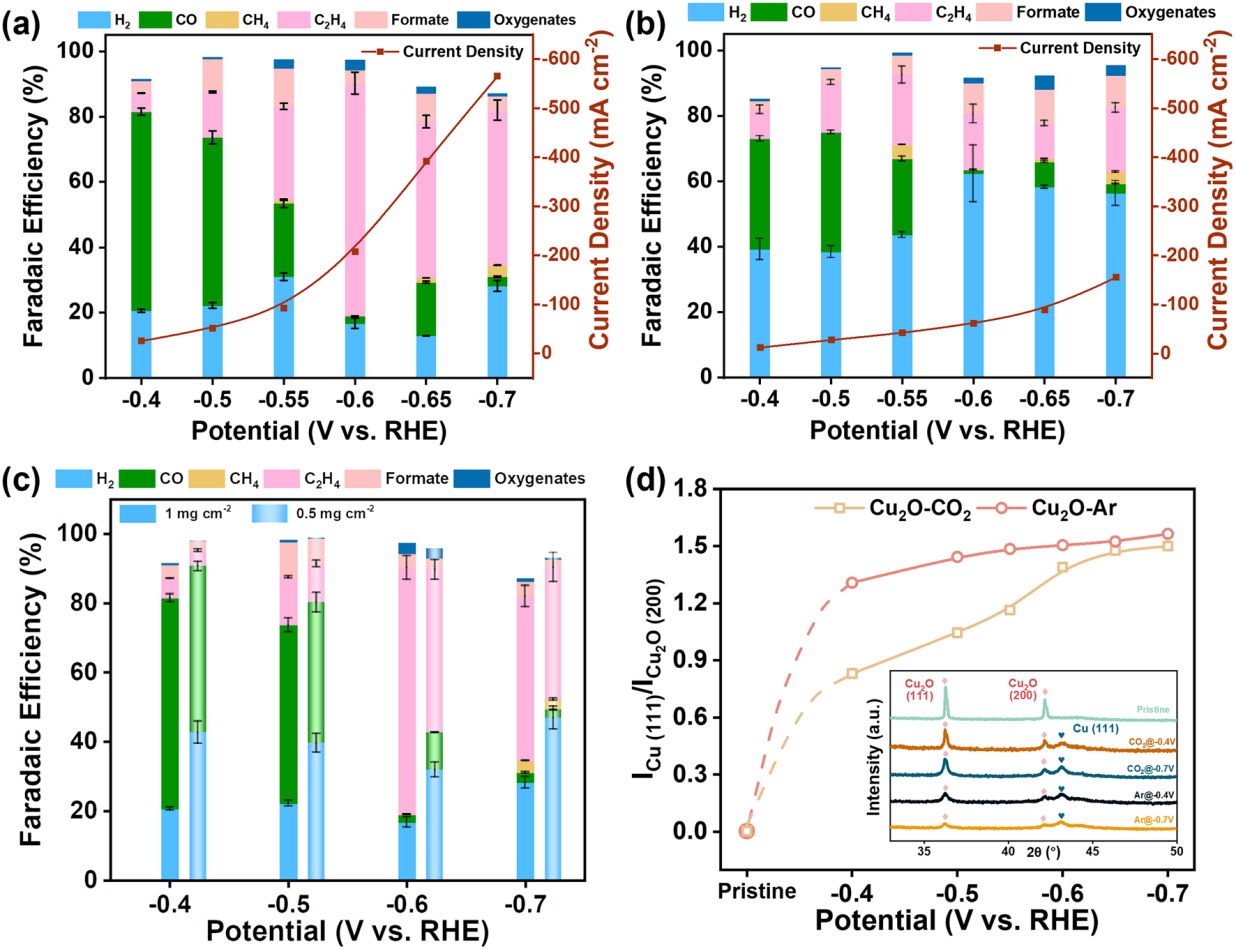
Electrochemical CO_2_RR performances and phase information of Cu_2_O nanocubes in a flow cell. Total current density and Faradaic efficiency of the main CO_2_RR products after activation in **a** CO_2_ and **b** Ar;** c** Faradaic efficiency of the main CO_2_RR products after activation with different loading; **d** XRD peak intensity ratio of Cu (111) to Cu_2_O (200), inset is typical XRD patterns after activation at different potentials of Cu_2_O-CO_2_ and Cu_2_O-Ar in a flow cell

The feeding gas-dependent reconstruction and catalytic performances were further probed in a flow cell with 1 M aqueous KOH (Fig. S16), aiming to assess the feasibility in catalytic environments close to practical application. Cu_2_O nanoparticles were loaded on gas diffusion electrodes (GDE, 1 cm^2^ with catalyst loading of 1 mg cm^−2^) to facilitate gas transportation for improved CO_2_ accessibility. Prior to electrochemical characterization, Cu_2_O nanocubes were activated at the potential of −0.6 V vs. RHE. As shown in Fig. 4a, b, the onset potential for CO_2_RR was negatively shifted by 200 mV and the maximum current density was increased by 2.5 times in comparison with those in H-cell for both catalysts (Fig. 3a–d). The apparent improvement in catalytic performances is ascribed to the change in cell configuration and electrolyte (from KHCO_3_ to KOH) [54]. More importantly, the feed gas-dependent catalytic performances are translated from H-cell to flow cell. In detail, Cu_2_O-CO_2_ exhibits a much better selectivity to CO_2_RR than Cu_2_O-Ar in the whole testing potential range. Once C_2_H_4_ is produced at an appreciable rate, the CO product becomes much more constrained. Akin to in H-cell, this indicates that CO intermediate (acting as the precursors of C_2_H_4_) plays a pivotal role in forming the passivating amorphous layer in the reconstruction process. At the optimal potential of −0.6 V, Cu_2_O-CO_2_ manifests an FE of 71% to C_2_H_4_ at the partial current density of −147.3 mA cm^−2^, whereas Cu_2_O-Ar shows an FE of only 17% at a much less partial current density of −10.5 mA cm^−2^. Moreover, we find that the selectivity to CO_2_RR is inferior at a lower loading of 0.5 mg cm^−2^ (Fig. 4c). For instance, the faradaic efficiency of C_2_H_4_ decreased by 24% at −0.6 V vs RHE. The inferior selectivity can be well rationalized by the *CO intermediate modulated reconstruction as proposed above. Catalysts with a decreasing loading lower the local intermediate concentration of *CO, thereby resulting in a reconstruction processed as in a CO_2_-deficient atmosphere (Ar) and leading to the production of HER active sites to inferior their selectivity toward C_2_H_4_. Similar catalyst density-dependent selectivity has been observed before, though it is attributed to the decreasing re-absorption of CO intermediates for CO_2_RR rather than for reconstruction [55, 56]. The result strongly supports our conclusion that the absorption of CO intermediate is crucial for reconstruction toward the amorphous layer that is favorable for the C–C bond coupling reaction. We further examined the influence of cell configuration and electrolytes on reconstruction. We conducted the pre-electrolysis and CO_2_RR electrolysis in a flow cell, using the same electrolyte as in the H-cell (0.5 M KHCO_3_). The resultant catalyst shows FE of C_2_H_4_ similar to that of Cu_2_O-CO_2_ in H-cell, ruling out the contribution to FE from cell configuration. It is also noteworthy that the difference in current density and potential should be ascribed to the cell configuration [54]. Following this, we did the pre-electrolysis in 0.5 M KHCO_3_ and then tested the CO_2_RR electrolysis in KOH electrolytes. The catalytic performances of the resultant catalyst are suppressed to some extent over that of Cu_2_O-CO_2_, but still much higher than that of Cu_2_O-Ar (Fig. S17). The result again confirms the dominating role of feeding gas rather than electrolytes in reconstruction.

To further evidence the passivation behavior, we investigated the potential dependent phase transition. XRD patterns of Cu_2_O-CO_2_ and Cu_2_O-Ar were recorded after being activated in the potential range of −0.4 ~ −0.7 V versus RHE with a decreasing interval of 0.1 or 0.05 V in Figs. 4d and S18. The peak intensity ratio of Cu-(111)/Cu_2_O-(200) is used to measure the amount of residual Cu_2_O. Besides, the typical XRD patterns of catalysts after pre-electrolysis at −0.4 and −0.7 V versus RHE were exhibited in the inset of Fig. 4d. The intensity ratio plotting against applied potentials shows that much more Cu_2_O was retained in Cu_2_O-CO_2_ than in Cu_2_O-Ar at potential higher than −0.65 V versus RHE. At the potential range of −0.4 to −0.6 V, the amount of metallic Cu is 7%–36% less in Cu_2_O-CO_2_. It suggests a much higher resistance of Cu_2_O-CO_2_ to reducing potential, agreeing well with the formation of a passivating amorphous layer under a CO_2_ atmosphere. When potentials are more negative than −0.6 V versus RHE, they exhibit similar conversion rates while distinct catalytic activity and selectivity. To reveal the underlying reason, we characterized the microstructures of Cu_2_O-CO_2_ and Cu_2_O-Ar after activating at the potential of −0.7 V (Fig. S18c-h). Cu_2_O-Ar retained the compact cubic shape, with Cu coated on inner Cu_2_O. In contrast, Cu_2_O-CO_2_ increased the transition of the amorphous part to Cu(0), leading to a quick increase in I_Cu(111)_/I_Cu2O(200)_. Importantly, we could still observe some residue amorphous part in Cu_2_O-CO_2_, which contributes to the higher catalytic performances (FE and current density) than that of Cu_2_O-Ar at the potential of −0.7 eV.

Though the feeding gas played a pivotal role in leading the selective CO_2_RR, we noted that it did not guarantee stable electrolysis for a period over 10 h in H-cell. To find the reason for the decay of performances, we trace the microstructure on the surface of Cu_2_O-CO_2_ in 3, 10, 20, and 30 h’ electrolysis (Fig. S9). (HR)TEM images reveal the progressive transition of the amorphous layer to Cu(0) nanoclusters. The particle size was getting larger with the prolonged electrolysis (Fig. S9c to S9f to S9i to S9l). Eventually, the surface transformed into a metallic state like Cu_2_O-Ar, exhibiting low catalytic activity in producing C_2_H_4_. This structure-performance correlation in the durability test underscores the essential role of the amorphous layer for selective CO_2_RR electrolysis.

### Mechanism of Feeding-Gas-Mediated Structural Evolution

The microstructural and electrochemical analysis above has shown the feeding gas plays a pivotal role in mediating the structure reconstruction and based on this we link the final structures to catalytic performances by identifying the catalytic active amorphous layer favorable for C–C bond coupling reaction. Given that *CO is regarded as an important intermediate for CO_2_RR and acts as the precursor for C_2_H_4_, we propose that *CO intermediate produced in the CO_2_ activation process could play a determining role in reconstruction, as well as the consequent high selectivity to C_2+_.

**Fig. 5:**
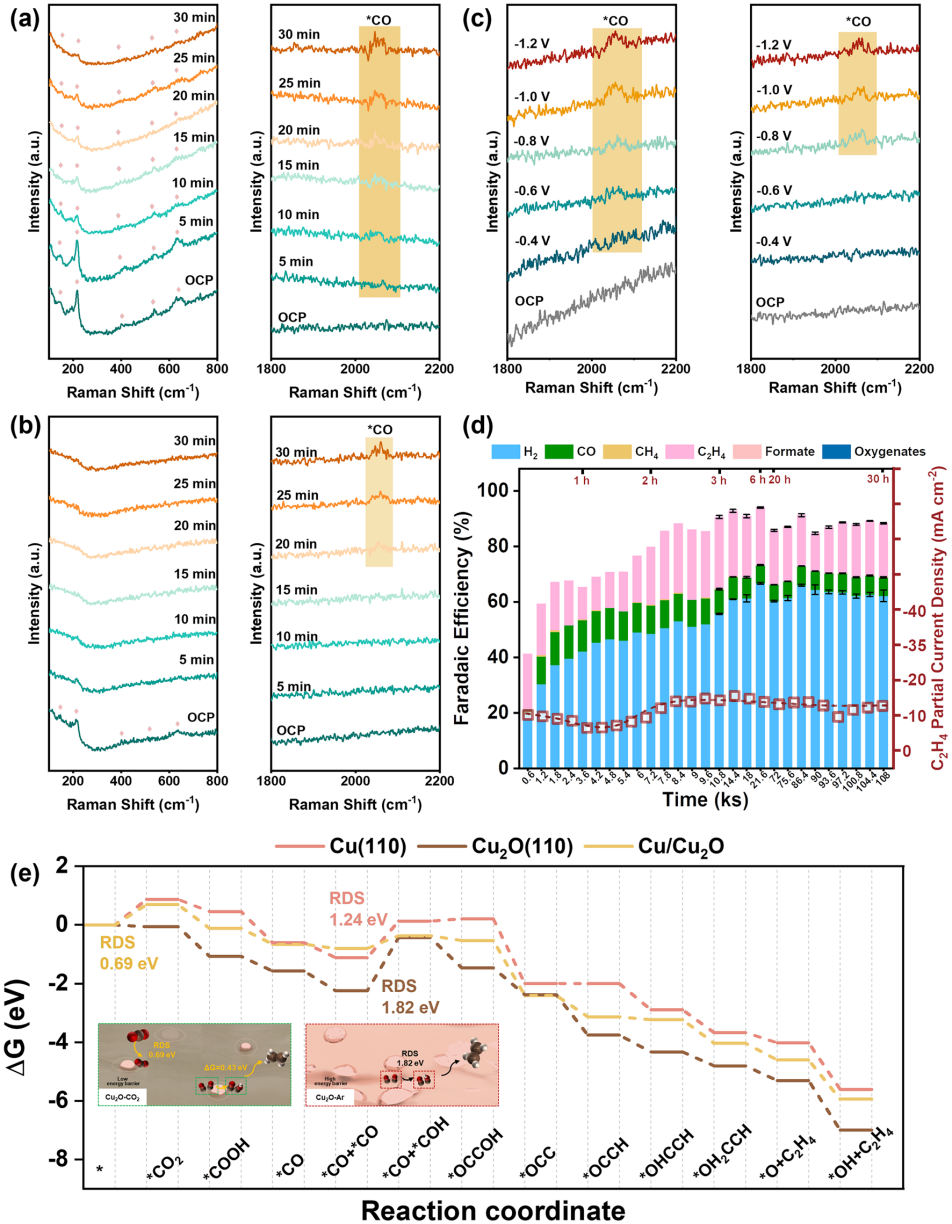
Mechanism of feeding-gas-mediated structural evolution. In situ Raman spectra evolution in a time scale with an interval of 5 min for **a** Cu_2_O-CO_2_ and **b** Cu_2_O-Ar in the pre-electrolysis process for activation. The applied potential is −1.1 V versus RHE;** c** In situ Raman spectra evolution with decreasing potential from OCP to −1.2 V versus RHE. The typical peaks of Cu_2_O are labeled with pink rhombus. The *CO intermediate is highlighted with an orange background. **d** Partial current density and Faradaic efficiency of the main CO_2_RR products after activation in O_2_. Red hollow squares are C_2_H_4_ partial current densities at each sampling time.** e** The reaction energy diagram for CO_2_RR to C_2_H_4_ on Cu (110) slab, Cu_2_O(110) slab, and Cu/Cu_2_O interface. (Color figure online)

To clarify the critical role of *CO intermediate, we performed in situ Raman analysis to directly probe the intermediate in the self-adaptive reconstruction process (Figs. 5a–c and S19-S22). The Raman spectra of Cu_2_O-CO_2_ were recorded every 5 min under electrolysis conditions in a CO_2_-saturated 0.5 M KHCO_3_ electrolyte. By contrast, the Cu_2_O-Ar catalyst was pretreated in an Ar atmosphere every 5 min and then transferred back to a CO_2_-saturated electrolyte to collect Raman spectra under electrolysis conditions. The peaks at 1026, 1353, and 1548 cm^−1^ are ascribed to the monodentate *CO_3_^2−^ (ν_1_CO_3_^2−^ of η_1_-CO_3_^2−^), bicarbonate, and bidentate carbonate (ν_as_CO_2_^−^ of η_2_(C,O)-CO_2_^−^), respectively (Figs. S19 and S20) [57]. Cu_2_O exhibits characteristic peaks at 142, 215, 405, 529, and 623 cm^−1^, corresponding to the 2Γ^–^_12_, 4Γ^–^_12_, Γ^+^_25_, and Γ^–^_12_ plus Γ^+^_25_ phonon modes. The peaks were weakened in prolonged pretreatment, in line with the transition from Cu_2_O to Cu(0) as revealed above [58]. To be noted, the transition is much slower in Cu_2_O-CO_2_. For instance, Cu_2_O is discernable even in 30 min of electrolysis for Cu_2_O-CO_2_, whereas it became insignificant in 10 min activation for Cu_2_O-Ar (Figs. 5a, b and S21). This observation is consistent with the passivation effect of the amorphous layer in protecting inner crystalline Cu_2_O nanoparticles from reduction. More interestingly, we observed an intermediate of *CO at a wavelength of around 2050 cm^−1^ (corresponding to νCO bands of *CO on atop sites) in only 5 min electrolysis in Cu_2_O-CO_2_, whereas it took more than 20 min to probe a distinguishable amount of *CO in Cu_2_O-Ar (Fig. 5a, b). This result validates the essential role of intermediate of *CO played in the formation of a catalytic active amorphous layer. Moreover, a much higher signal of *CO intermediate is present in Cu_2_O-CO_2_, corresponding well with the larger amount of Cu(I) in the amorphous layer.

To confirm the role of the intermediate of *CO in reconstruction, the driving force was modulated by varying the applied potential in the range from OCP to –1.2 V versus RHE with an interval of 0.2 V. The Raman spectra were recorded at each potential to analyze the phase and intermediate transition (Fig. 5c). The increasing driving force pushes forward the phase transition at more negative potentials (Fig. S22). Notably, the transition degree is slower in Cu_2_O-CO_2_ than in Cu_2_O-Ar, in line with the passivation effect of the amorphous layer as revealed above. Moreover, Cu_2_O-CO_2_ started to showcase the signal of *CO intermediate at a potential of −0.6 V versus RHE, which is around 0.2 V more positive than Cu_2_O-Ar. Meanwhile, the signals were more evident in Cu_2_O-CO_2_ over in Cu_2_O-Ar at each potential. The higher onset potential and higher intensity of the Raman signal indicate that *CO intermediate is responsible for the formation of Cu(I)-rich amorphous layer, which in turn contributes to the better selectivity for value-added C_2_H_4_.

To validate the role of *CO intermediate, we changed the feeding gas to pure CO for activation in the pre-electrolysis (Fig. S23a-d). The product (denoted as Cu_2_O-CO) shares the same catalytic performances (total current density and Faradaic efficiency) as that of Cu_2_O-CO_2_ (Fig. S23a-d), confirming the essential role of *CO intermediate in producing an amorphous layer. This is further validated by the similar FE of C_2_H_4_ in electrocatalytic CORR (Fig. S23e, f). We further changed the feeding gas to a mixture of CO/CO_2_ (volume ratio: 1:1). It again exhibits the same catalytic performance. These data suggest a few CO are enough to drive the transition from crystalline Cu_2_O to the amorphous Cu_x_O layer. The in situ formed *CO could be favorable, as CO is released near the active sites and results in a higher local concentration than the feeding CO externally.

To confirm the mediating process of reaction intermediate in structural evolution, we shift the gas from CO_2_ to O_2_, because oxygen-related intermediates (for instance, *OH, *O, and/or *OOH) could absorb on Cu-based catalysts in electrocatalytic oxygen reduction reaction (ORR) or cocatalysis of ORR and CO_2_RR [59, 60]. Akin to the above protocol in Ar/CO_2_, Cu_2_O Cubes were imposed to a potential of −1.1 V versus RHE in pure O_2_ atmosphere for 2000 s for reconstruction, and then subjected to electrocatalytic CO_2_RR. It turned out that the resulting catalysts exhibited in-between total current densities and partial current densities and FE of C_2_H_4_ (−59.6 and −15.4 mA cm^−2^, and 26%, Figs. 5d and S24). The potential dependent catalytic activity and selectivity were further evaluated for Cu_2_O-O_2_ (Fig. S25). The trend is the same as that of Cu_2_O-Ar and Cu_2_O-CO_2_. The FE of C_2_H_4_ increased at the potential where the FE of CO decreased, and the more negative of potential, the larger the FE ratio of C_2_H_4_ to CO. This result supports our conclusion that the reaction intermediate mediates the structural evolution and selectivity. Also, there are some inconsistencies between Cu_2_O-O_2_ and Cu_2_O-CO_2_/Cu_2_O-Ar. Firstly, Cu_2_O-O_2_ seems more stable than Cu_2_O-CO_2_, despite the lower catalytic activity (Figs. S24 and S26). The better durability should be due to the production of extra CO, which could supply abundant *CO intermediates to stabilize Cu(I) active sites for the C–C bond coupling reaction. This attribution agrees well with the fact that weak durability corresponds to the depletion of CO product at the potential applied for durability test. Secondly, the optimal FE of C_2_H_4_ shifts to a more positive potential of −1.0 V versus RHE (Fig. S25). The change could be due to the distinct absorption of intermediates and the different surface electronic structures, which are under further investigation.

Furthermore, we examined the effect of pre-electrolysis in mixed CO_2_/O_2_ with a volume ratio varying from 3:1, 1:1, to 1:3 (Fig. S27). When the ratio is equal to or larger than 1:1, the electrocatalysts behave like Cu_2_O-CO_2_, showing higher FE and partial current density in producing C_2_H_4_. When it is less than 1:1, the electrocatalysts behave like Cu_2_O-O_2_, exhibiting suppressed FE but improved durability (Fig. S27). This is in line with the stronger absorption of *CO than oxygen-related intermediates (*OH, *O, and/or *OOH), further confirming the essential role of the intermediate of *CO in the resulting catalyst surface favorable for selective C_2_H_4_ production. Previous studies have also shown that *CO intermediate has a strong absorption ability to Cu-based materials, and O-related intermediates have a moderate absorption ability, whereas Ar is relatively inert to absorption on any substrates. The absorption ability of gas molecules and/or the corresponding intermediates correlates well with the surface reconstruction and the resulting catalytic performances, supporting the critical role of the reaction intermediate in structural reconstruction. More importantly, our finding could imply a new dimension to activate precatalysts for CO_2_RR that is to control the components of feeding gases for activation. Indeed, previous work has shown the unique selectivity in co-feeding gases of O_2_ and CO_2_ [59]. Though they rationalized it by the concept of co-electrolysis involving intermediates of *OH, the feeding-gas resultant reconstruction cannot be ruled out.

We performed density functional (DFT) theory calculations to further specify the essential role of *CO in the resulting catalyst surface that is selective for C_2_H_4_ production. Since the reconstruction is along with CO_2_RR, we calculated the change of Gibbs energy for intermediates adsorption on Cu(110) slab, Cu_2_O(110) slab, and the interface of Cu(110)/Cu_2_O(110) (Figs. 5e and S28-S31). Cu(110) and Cu_2_O(110) share the same rate-determining step (RDS) of *CO + *CO → *CO + *COH, whereas the Cu(110)/Cu_2_O(110) has RDS of CO_2_ activation (* + CO_2_ → *CO_2_). The energy barrier in RDS is only 0.69 eV for the Cu(110)/Cu_2_O(110) interface, significantly lower than that for Cu(110) (1.24 eV) and Cu_2_O (110) (1.82 eV). This is consistent with the outstanding CO_2_RR performance of Cu_2_O-CO_2_, possessing a large amount of Cu/Cu_2_O interface. The high energy barrier of Cu(110) and Cu_2_O(110) indicates the surface is mostly covered by *CO, in line with our in situ Raman spectra. Furthermore, we calculated the absorption enthalpy of *CO. Cu_2_O(110) exhibits the lowest absorption enthalpy of −1.09 eV. The low absorption enthalpy of Cu_2_O(110) is in line with the strong and stable absorption of *CO in Cu_2_O-CO_2_. Once Cu_2_O was reduced to Cu(0), the absorption enthalpy increased to −0.13 eV on surfaces and to −0.20 eV on Cu(110)/Cu_2_O(110) interfaces. We noted that the absorption enthalpy of Cu(110)/Cu_2_O(110) interfaces is lower than Cu(0), suggesting that Cu(110)/Cu_2_O(110) interfaces mitigate the further reduction of the catalysts to metallic copper.

In the previous literature, Cu_2_O has been well investigated, given the superb catalytic performances in producing C_2_H_4_ [40, 42, 43]. The structural evolution was revealed and the driving force was ascribed to the applied negative potential solely. Though CO_2_ has been found to work for modulating the crystal growth, cation leaching, and morphology evolution of copper [38–41], it has rarely been reported to regulate the reconstruction of Cu_2_O. Different from those focusing on the catalyst itself, this work took the gas accessibility into account. The new findings provide additional information to elucidate previous controversial results and rationalize the debates on catalytic mechanisms [44, 45]. This work also highlights the importance of the microstructure design of catalysts. The modulation of microenvironments including gas accessibility, local pH, and triple-phase interface not only promotes catalytic reaction as most works suggested before [36, 37] but also guides the catalyst reconstruction toward favorable active structures as found here.

### Knowledge Guiding Design of Selective and Durable CO_2_RR Catalysts for Value-Added Products of C_2_H_4_

**Fig. 6:**
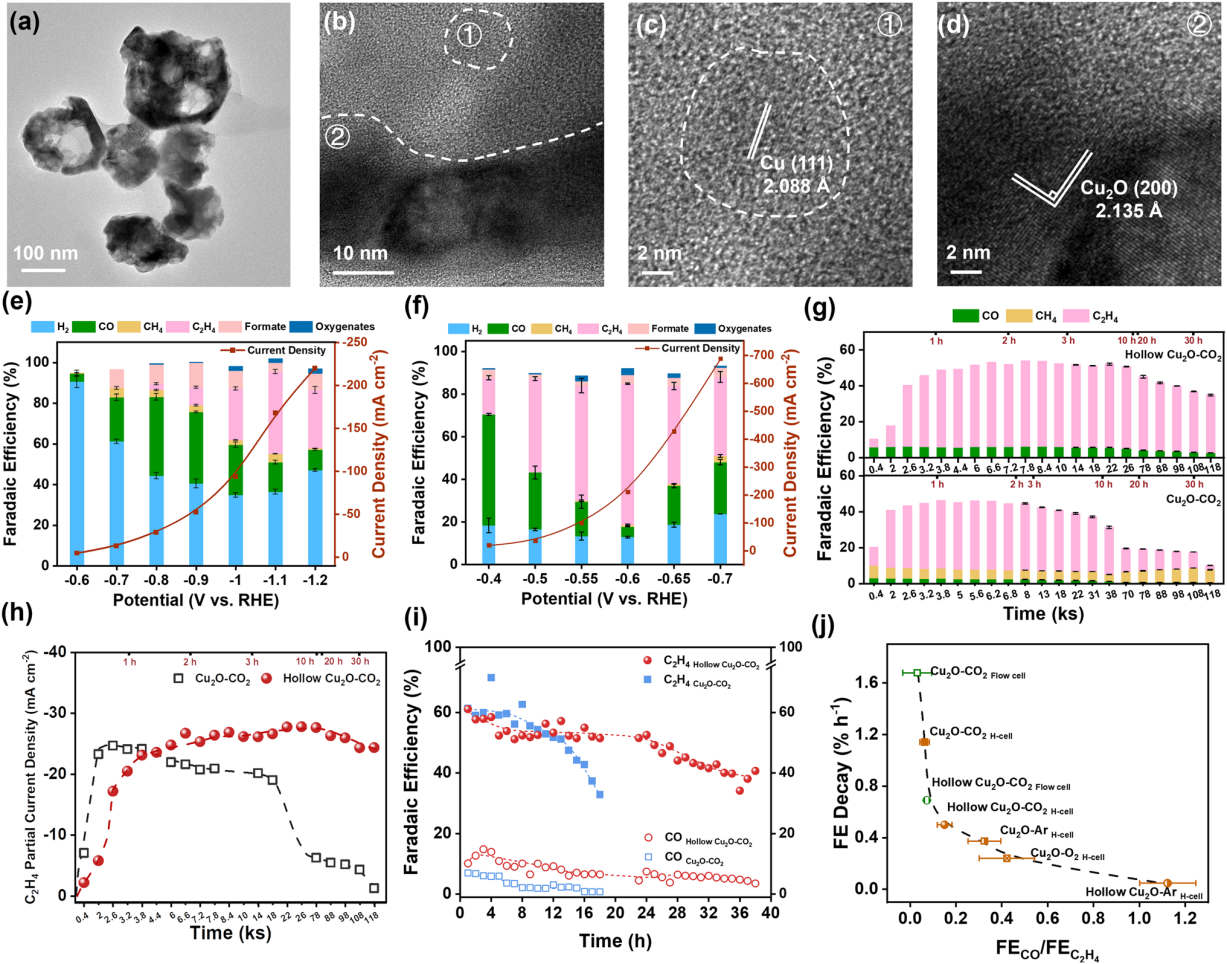
Microstructural evolution and electrochemical CO_2_RR performances of Cu_2_O hollow spheres in an H-cell and a flow cell.** a** TEM images and **b-d** HRTEM images of Cu_2_O hollow spheres after activation in CO_2_ atmosphere for 2000 s. The dashed line in **b** is the border between crystalline Cu_2_O and amorphous Cu_x_O. The c is the enlarged region of ① in **b** and the **d** is the enlarged region of ② in **b**; **e** Total current density and Faradaic efficiency of the main CO_2_RR products plotting against applied potentials after activation in an H-cell; **f** Total current density and Faradaic efficiency of the main CO_2_RR products plotting against applied potentials after activation in a flow-cell; **g** Faradaic efficiency and **h** partial current density of the main CO_2_RR products of Cu_2_O hollow spheres and solid Cu_2_O nanocubes after activation in CO_2_. The applied potential is −1.1 V versus RHE for electrolysis in an H-cell; **i** Stability of hollow Cu_2_O-CO_2_, Cu_2_O-CO_2,_ and Cu_2_O-Ar at the current density of −200 mA cm^−2^ in a flow-cell; **j** Correlation between FE decay rate and FE_CO_/FE_C2H4_ ratio during the stability tests

Besides catalytic activity and selectivity, it appears that the dynamic reconstruction also affects the durability. The partial current density for C_2_H_4_ started to decay for Cu_2_O-CO_2_ once it achieved the highest FE and partial current density of C_2_H_4_ in both an H-cell and a flow cell (Figs. 3a and S32). The FE of C_2_H_4_ decreases by 35.6% in 31 h in the H-cell and 28.5% in 18 h in the flow cell (Fig. S32). The above analysis has shown that the absorption of *CO intermediate is crucial for the formation and stability of the catalytic active amorphous layer. In particular, it stabilizes Cu(I) sites favorable for C–C bond coupling reaction. Given that *CO intermediate is the precursor of C_2_H_4_ and rare CO product was released at the potential for stability assessment (as revealed in Figs. 3a, b and 5d), we suggest that the decay could be attributed to the fact that active Cu(I) sites were destabilized by the depletion of *CO intermediates for the production of C_2_H_4_. This is supported by the much better durability of Cu_2_O-O_2_, which exhibited a CO FE of up to 12% (Fig. 5d). To further validate it, we excavated Cu_2_O nanoparticles to form hollow nanospheres (Figs. S33 and S34), aiming to increase the accessibility of CO_2_ to produce more CO intermediates and confine them inside to maintain a CO-rich environment to stabilize Cu(I). Activation in a CO_2_-sufficient atmosphere did not change the hollow morphology but resulted in amorphous layers encapsulating on crystalline Cu_2_O nanoparticles (Fig. 6a–d). Analogous to Cu_2_O-CO_2_, well-dispersed tiny Cu(0) nanoparticles of 10 nm in size were present in the amorphous layer. The catalytic performances were then assessed in both H-cell (in the range from −0.6 to −1.2 V versus RHE, Fig. 6e) and flow-cell (in the range from −0.4 to −0.7 V versus RHE, Fig. 6f). Cu_2_O hollow nanospheres exhibited the similar catalytic performances (including current densities and FE of various products) as Cu_2_O-CO_2_. The significant difference between them is that more CO products were released for hollow nanospheres at the optimal potential (the one for the stability test). The FE of CO is 14.5% in H-cell and 4.8% in flow-cell for Cu_2_O hollow nanospheres, 2–6 times higher than that for Cu_2_O-CO_2_ (2.4%). As expected, Cu_2_O hollow nanospheres exhibited improved durability than Cu_2_O-CO_2_. In a H-cell, Cu_2_O hollow nanospheres maintained a C_2_H_4_ partial current density of around −26 mA cm^−2^ with a FE of 47% in > 30 h electrolysis, whereas Cu_2_O-CO_2_ lost the FE by 15.3% (Fig. 6g) and partial current density by 9% (Fig. 6h). In a flow cell, the durability was measured at a practical current density of 200 mA cm^−2^ (Figs. 6i and S35-S36). The FE of Cu_2_O hollow nanospheres decreased at a decay rate of 0.53% h^−1^ in 38 h of electrolysis, one-third of that of Cu_2_O-CO_2_ (1.58% h^−1^ in 18 h of electrolysis). The superb activity, selectivity, and durability outperform Cu-based electrocatalysts reported previously (Table S4). More interestingly, we find that durability described by the decay rate correlates with the FE ratio of CO/C_2_H_4_ (Figs. 6j and S37-S38). It shows that a larger proportion of CO corresponds to better stability, agreeing well with the role of CO intermediates in stabilizing Cu(I) for a favorable C–C bond coupling reaction. Our finding also rationalizes the durable and selective production of C_2_H_4_ for low-dimensional Cu_2_O catalysts [27].

## Conclusion

In conclusion, our work uncovered the essential role of feeding gas (CO_2_ accessibility) on structural reconstruction and further elucidated how it affected the catalytic performances, by recognizing a CO_2_-induced passivation process in structural reconstruction and a self-stabilizing process in catalytic production of C_2_H_4_. The CO_2_-rich atmosphere led to a catalyst surface favorable for CO_2_RR, whereas the CO_2_-deficient one (Ar) preferred that for hydrogen evolution reaction (HER), exhibiting a ~ 4 times difference in ethylene (C_2_H_4_) Faradaic efficiency (FE) and ~ 8 times difference in current densities. The *CO intermediates played a pivotal role in stabilizing Cu(I) sites, leading to the formation of a reduction-resistant but catalytic active amorphous layer in the reconstruction process. Furthermore, we found extra CO production was indispensable for the robust production of C_2_H_4_. An inverse correlation between durability and FE_CO_/FE_C2H4_ was disclosed. We attributed it to the self-stabilization of Cu(I) sites in the CO atmosphere. Taking advantage of this knowledge, we fabricated hollow Cu_2_O nanospheres and demonstrated durable electrolysis for over 48 h at a current density of −200 mA cm^−2^ in producing C_2_H_4_ with an FE of up to 61% at −0.6 V_RHE_ in a flow cell. Our work recognizes the previously overlooked passivation reconstruction and self-stabilizing behavior and highlights the critical role of the local atmosphere in modulating the reconstruction behavior and catalytic process, serving as an important supplement in understanding and designing high-performance CO_2_RR (pre)catalysts. A hybrid catalyst is therefore suggested, with one component producing *CO to increase the local CO concentration for favorable reconstruction and durable catalysis. Additionally, the strong link between atmosphere and structural reconstruction has an implication for the design of precatalysts and the revival of degraded catalysts.

## Supplementary Information

Below is the link to the electronic supplementary material.

Supplementary file1 (DOCX 17496 KB)

## References

[1] J.H. Montoya, L.C. Seitz, P. Chakthranont, A. Vojvodic, T.F. Jaramillo et al., Materials for solar fuels and chemicals. Nat. Mater. **16**, 70–81 (2017). 10.1038/nmat4778 10.1038/nmat4778 27994241

[2] P. De Luna, C. Hahn, D. Higgins, S.A. Jaffer, T.F. Jaramillo et al., What would it take for renewably powered electrosynthesis to displace petrochemical processes? Science **364**, eaav3506 (2019). 10.1126/science.aav3506 31023896 10.1126/science.aav3506

[3] D. Gao, R.M. Arán-Ais, H.S. Jeon, B. Roldan Cuenya, Rational catalyst and electrolyte design for CO_2_ electroreduction towards multicarbon products. Nat. Catal. **2**, 198–210 (2019). 10.1038/s41929-019-0235-5

[4] S. Nitopi, E. Bertheussen, S.B. Scott, X. Liu, A.K. Engstfeld et al., Progress and perspectives of electrochemical CO_2_ reduction on copper in aqueous electrolyte. Chem. Rev. **119**, 7610–7672 (2019). 10.1021/acs.chemrev.8b00705 31117420 10.1021/acs.chemrev.8b00705

[5] Y. Wang, J. Liu, G. Zheng, Designing copper-based catalysts for efficient carbon dioxide electroreduction. Adv. Mater. **33**, 2005798 (2021). 10.1002/adma.202005798 10.1002/adma.202005798 33913569

[6] W. Ma, X. He, W. Wang, S. Xie, Q. Zhang et al., Electrocatalytic reduction of CO_2_ and CO to multi-carbon compounds over Cu-based catalysts. Chem. Soc. Rev. **50**, 12897–12914 (2021). 10.1039/d1cs00535a 34609390 10.1039/d1cs00535a

[7] G.L. De Gregorio, T. Burdyny, A. Loiudice, P. Iyengar, W.A. Smith et al., Facet-dependent selectivity of Cu catalysts in electrochemical CO_2_ reduction at commercially viable current densities. ACS Catal. **10**, 4854–4862 (2020). 10.1021/acscatal.0c00297 32391186 10.1021/acscatal.0c00297 PMC7199425

[8] Z.-Z. Niu, F.-Y. Gao, X.-L. Zhang, P.-P. Yang, R. Liu et al., Hierarchical copper with inherent hydrophobicity mitigates electrode flooding for high-rate CO_2_ electroreduction to multicarbon products. J. Am. Chem. Soc. **143**, 8011–8021 (2021). 10.1021/jacs.1c01190 33913717 10.1021/jacs.1c01190

[9] S. Kong, X. Lv, X. Wang, Z. Liu, Z. Li et al., Delocalization state-induced selective bond breaking for efficient methanol electrosynthesis from CO_2_. Nat. Catal. **6**, 6–15 (2022). 10.1038/s41929-022-00887-z

[10] Y. Xue, P. Wang, M. He, T. Zhang, C. Yang et al., Rare earth nanomaterials in electrochemical reduction of carbon dioxide. Coord. Chem. Rev. **516**, 215983 (2024). 10.1016/j.ccr.2024.215983

[11] D.-H. Nam, P. De Luna, A. Rosas-Hernández, A. Thevenon, F. Li et al., Molecular enhancement of heterogeneous CO_2_ reduction. Nat. Mater. **19**, 266–276 (2020). 10.1038/s41563-020-0610-2 32099112 10.1038/s41563-020-0610-2

[12] C.W. Li, M.W. Kanan, CO_2_ reduction at low overpotential on Cu electrodes resulting from the reduction of thick Cu_2_O films. J. Am. Chem. Soc. **134**, 7231–7234 (2012). 10.1021/ja3010978 22506621 10.1021/ja3010978

[13] K.P. Kuhl, E.R. Cave, D.N. Abram, T.F. Jaramillo, New insights into the electrochemical reduction of carbon dioxide on metallic copper surfaces. Energy Environ. Sci. **5**, 7050–7059 (2012). 10.1039/C2EE21234J

[14] K. Jiang, R.B. Sandberg, A.J. Akey, X. Liu, D.C. Bell et al., Metal ion cycling of Cu foil for selective C-C coupling in electrochemical CO_2_ reduction. Nat. Catal. **1**, 111–119 (2018). 10.1038/s41929-017-0009-x

[15] S.Y. Lee, S.Y. Chae, H. Jung, C.W. Lee, N. Le Tri et al., Controlling the C2+ product selectivity of electrochemical CO_2_ reduction on an electrosprayed Cu catalyst. J. Mater. Chem. A **8**, 6210–6218 (2020). 10.1039/C9TA13173F

[16] G. Liu, M. Lee, S. Kwon, G. Zeng, J. Eichhorn et al., CO_2_ reduction on pure Cu produces only H_2_ after subsurface O is depleted: theory and experiment. Proc. Natl. Acad. Sci. U.S.A. **118**, e2012649118 (2021). 10.1073/pnas.2012649118 34083432 10.1073/pnas.2012649118 PMC8201769

[17] F. Dattila, R. García-Muelas, N. López, Active and selective ensembles in oxide-derived copper catalysts for CO_2_ reduction. ACS Energy Lett. **5**, 3176–3184 (2020). 10.1021/acsenergylett.0c01777

[18] C.W. Li, J. Ciston, M.W. Kanan, Electroreduction of carbon monoxide to liquid fuel on oxide-derived nanocrystalline copper. Nature **508**, 504–507 (2014). 10.1038/nature13249 24717429 10.1038/nature13249

[19] Y. Lum, J.W. Ager, Evidence for product-specific active sites on oxide-derived Cu catalysts for electrochemical CO_2_ reduction. Nat. Catal. **2**, 86–93 (2019). 10.1038/s41929-018-0201-7

[20] D. Zhong, Z.-J. Zhao, Q. Zhao, D. Cheng, B. Liu et al., Coupling of Cu(100) and (110) facets promotes carbon dioxide conversion to hydrocarbons and alcohols. Angew. Chem. Int. Ed. **60**, 4879–4885 (2021). 10.1002/anie.202015159 10.1002/anie.202015159 33231928

[21] H. Li, T. Liu, P. Wei, L. Lin, D. Gao et al., High-rate CO_2_ electroreduction to C_2+_ products over a copper-copper iodide catalyst. Angew. Chem. Int. Ed. **60**, 14329–14333 (2021). 10.1002/anie.202102657 10.1002/anie.202102657 33837619

[22] R.M. Arán-Ais, F. Scholten, S. Kunze, R. Rizo, B. Roldan Cuenya, The role of *in situ* generated morphological motifs and Cu(I) species in C_2+_ product selectivity during CO_2_ pulsed electroreduction. Nat. Energy **5**, 317–325 (2020). 10.1038/s41560-020-0594-9

[23] T.-C. Chou, C.-C. Chang, H.-L. Yu, W.-Y. Yu, C.-L. Dong et al., Controlling the oxidation state of the Cu electrode and reaction intermediates for electrochemical CO_2_ reduction to ethylene. J. Am. Chem. Soc. **142**, 2857–2867 (2020). 10.1021/jacs.9b11126 31955572 10.1021/jacs.9b11126

[24] P. De Luna, R. Quintero-Bermudez, C.-T. Dinh, M.B. Ross, O.S. Bushuyev et al., Catalyst electro-redeposition controls morphology and oxidation state for selective carbon dioxide reduction. Nat. Catal. **1**, 103–110 (2018). 10.1038/s41929-017-0018-9

[25] Y. Zhou, F. Che, M. Liu, C. Zou, Z. Liang et al., Dopant-induced electron localization drives CO_2_ reduction to C_2_ hydrocarbons. Nat. Chem. **10**, 974–980 (2018). 10.1038/s41557-018-0092-x 30013194 10.1038/s41557-018-0092-x

[26] X. Xia, Y. Wang, A. Ruditskiy, Y. Xia, 25th anniversary article: galvanic replacement: a simple and versatile route to hollow nanostructures with tunable and well-controlled properties. Adv. Mater. **25**, 6313–6333 (2013). 10.1002/adma.201302820 24027074 10.1002/adma.201302820

[27] P. Wang, S. Meng, B. Zhang, M. He, P. Li et al., Sub-1 nm Cu_2_O nanosheets for the electrochemical CO_2_ reduction and valence state-activity relationship. J. Am. Chem. Soc. **145**, 26133–26143 (2023). 10.1021/jacs.3c08312 37977134 10.1021/jacs.3c08312

[28] H. Zhang, Y. Wang, Q. Lei, Y. Wang, C. Tang et al., Optimizing Cu^+^-Cu^0^ synergy by operando tracking of Cu_2_O nanocatalysts during the electrochemical CO_2_ reduction reaction. Nano Energy **118**, 108920 (2023). 10.1016/j.nanoen.2023.108920

[29] X. Tan, K. Sun, Z. Zhuang, B. Hu, Y. Zhang et al., Stabilizing copper by a reconstruction-resistant atomic Cu-O-Si interface for electrochemical CO_2_ reduction. J. Am. Chem. Soc. **145**, 8656–8664 (2023). 10.1021/jacs.3c01638 10.1021/jacs.3c01638 37029738

[30] Y. Cao, S. Chen, S. Bo, W. Fan, J. Li et al., Single atom Bi decorated copper alloy enables C-C coupling for electrocatalytic reduction of CO_2_ into C_2+_ products. Angew. Chem. Int. Ed. **62**, e202303048 (2023). 10.1002/anie.202303048 10.1002/anie.202303048 37249478

[31] L. Xu, X. Ma, L. Wu, X. Tan, X. Song et al., *In situ* periodic regeneration of catalyst during CO_2_ electroreduction to C_2+_ products. Angew. Chem. Int. Ed. **61**, e202210375 (2022). 10.1002/anie.202210375 10.1002/anie.202210375 35876024

[32] B. Liu, X. Yao, Z. Zhang, C. Li, J. Zhang et al., Synthesis of Cu_2_O nanostructures with tunable crystal facets for electrochemical CO_2_ reduction to alcohols. ACS Appl. Mater. Interfaces **13**, 39165–39177 (2021). 10.1021/acsami.1c03850 34382393 10.1021/acsami.1c03850

[33] Z.-Z. Wu, X.-L. Zhang, Z.-Z. Niu, F.-Y. Gao, P.-P. Yang et al., Identification of Cu(100)/Cu(111) interfaces as superior active sites for CO dimerization during CO_2_ electroreduction. J. Am. Chem. Soc. **144**, 259–269 (2022). 10.1021/jacs.1c09508 34962375 10.1021/jacs.1c09508

[34] B. Deng, M. Huang, K. Li, X. Zhao, Q. Geng et al., The crystal plane is not the key factor for CO_2_-to-methane electrosynthesis on reconstructed Cu_2_O microparticles. Angew. Chem. Int. Ed. **61**, e202114080 (2022). 10.1002/anie.202114080 10.1002/anie.202114080 34882934

[35] L. Zaza, K. Rossi, R. Buonsanti, Well-defined copper-based nanocatalysts for selective electrochemical reduction of CO_2_ to C_2_ products. ACS Energy Lett. **7**, 1284–1291 (2022). 10.1021/acsenergylett.2c00035

[36] Y. Lin, T. Wang, L. Zhang, G. Zhang, L. Li et al., Tunable CO_2_ electroreduction to ethanol and ethylene with controllable interfacial wettability. Nat. Commun. **14**, 3575 (2023). 10.1038/s41467-023-39351-2 37328481 10.1038/s41467-023-39351-2 PMC10275897

[37] P.-P. Yang, M.-R. Gao, Enrichment of reactants and intermediates for electrocatalytic CO_2_ reduction. Chem. Soc. Rev. **52**, 4343–4380 (2023). 10.1039/d2cs00849a 37318005 10.1039/d2cs00849a

[38] Y. Wang, Z. Wang, C.-T. Dinh, J. Li, A. Ozden et al., Catalyst synthesis under CO_2_ electroreduction favours faceting and promotes renewable fuels electrosynthesis. Nat. Catal. **3**, 98–106 (2019). 10.1038/s41929-019-0397-1

[39] W.T. Osowiecki, J.J. Nussbaum, G.A. Kamat, G. Katsoukis, M. Ledendecker et al., Factors and dynamics of Cu nanocrystal reconstruction under CO_2_ reduction. ACS Appl. Energy Mater. **2**, 7744–7749 (2019). 10.1021/acsaem.9b01714

[40] Q. Ren, N. Zhang, Z. Dong, L. Zhang, X. Chen et al., Structural evolution of Cu_2_O nanocube electrocatalysts for the CO_2_ reduction reaction. Nano Energy **106**, 108080 (2023). 10.1016/j.nanoen.2022.108080

[41] Z.-Z. Niu, L.-P. Chi, Z.-Z. Wu, P.-P. Yang, M.-H. Fan et al., CO_2_-assisted formation of grain boundaries for efficient CO-CO coupling on a derived Cu catalyst. Natl. Sci. Open **2**, 20220044 (2023). 10.1360/nso/20220044

[42] P. Grosse, A. Yoon, C. Rettenmaier, A. Herzog, S.W. Chee et al., Dynamic transformation of cubic copper catalysts during CO_2_ electroreduction and its impact on catalytic selectivity. Nat. Commun. **12**, 6736 (2021). 10.1038/s41467-021-26743-5 34795221 10.1038/s41467-021-26743-5 PMC8602378

[43] Y. Yang, S. Louisia, S. Yu, J. Jin, I. Roh et al., operando studies reveal active Cu nanograins for CO_2_ electroreduction. Nature **614**, 262–269 (2023). 10.1038/s41586-022-05540-0 36755171 10.1038/s41586-022-05540-0

[44] X. Wang, K. Klingan, M. Klingenhof, T. Möller, J. Ferreira de Araújo et al., Morphology and mechanism of highly selective Cu(II) oxide nanosheet catalysts for carbon dioxide electroreduction. Nat. Commun. **12**, 794 (2021). 10.1038/s41467-021-20961-7 33542208 10.1038/s41467-021-20961-7 PMC7862240

[45] J. Huang, N. Hörmann, E. Oveisi, A. Loiudice, G.L. De Gregorio et al., Potential-induced nanoclustering of metallic catalysts during electrochemical CO_2_ reduction. Nat. Commun. **9**, 3117 (2018). 10.1038/s41467-018-05544-3 30082872 10.1038/s41467-018-05544-3 PMC6079067

[46] W. Liu, P. Zhai, A. Li, B. Wei, K. Si et al., Electrochemical CO_2_ reduction to ethylene by ultrathin CuO nanoplate arrays. Nat. Commun. **13**, 1877 (2022). 10.1038/s41467-022-29428-9 35387994 10.1038/s41467-022-29428-9 PMC8986799

[47] L. Laffont, M.Y. Wu, F. Chevallier, P. Poizot, M. Morcrette et al., High resolution EELS of Cu–V oxides: application to batteries materials. Micron **37**, 459–464 (2006). 10.1016/j.micron.2005.11.007 16376088 10.1016/j.micron.2005.11.007

[48] V.J. Keast, A.J. Scott, R. Brydson, D.B. Williams, J. Bruley, Electron energy-loss near-edge structure–a tool for the investigation of electronic structure on the nanometre scale. J. Microsc. **203**, 135–175 (2001). 10.1046/j.1365-2818.2001.00898.x 11489072 10.1046/j.1365-2818.2001.00898.x

[49] W. Li, C. Ni, Electron energy loss spectroscopy (EELS). *Encyclopedia of Tribology*. Springer US, (2013), pp. 940–945. 10.1007/978-0-387-92897-5_1223

[50] H. Luo, B. Li, J.-G. Ma, P. Cheng, Surface modification of nano-Cu_2_O for controlling CO_2_ electrochemical reduction to ethylene and syngas. Angew. Chem. Int. Ed. **61**, e202116736 (2022). 10.1002/anie.202116736 10.1002/anie.202116736 34995001

[51] Q. Wu, R. Du, P. Wang, G.I.N. Waterhouse, J. Li et al., Nanograin-boundary-abundant Cu_2_O-Cu nanocubes with high C_2+_ selectivity and good stability during electrochemical CO_2_ reduction at a current density of 500 mA/cm^2^. ACS Nano **17**, 12884–12894 (2023). 10.1021/acsnano.3c04951 37339159 10.1021/acsnano.3c04951

[52] J. Feng, L. Wu, S. Liu, L. Xu, X. Song et al., Improving CO_2_-to-C_2+_ product electroreduction efficiency *via* atomic lanthanide dopant-induced tensile-strained CuO_*x*_ catalysts. J. Am. Chem. Soc. **145**, 9857–9866 (2023). 10.1021/jacs.3c02428 37092347 10.1021/jacs.3c02428

[53] B. Cao, F.-Z. Li, J. Gu, Designing Cu-based tandem catalysts for CO_2_ electroreduction based on mass transport of CO intermediate. ACS Catal. **12**, 9735–9752 (2022). 10.1021/acscatal.2c02579

[54] D.M. Weekes, D.A. Salvatore, A. Reyes, A. Huang, C.P. Berlinguette, Electrolytic CO_2_ reduction in a flow cell. Acc. Chem. Res. **51**, 910–918 (2018). 10.1021/acs.accounts.8b00010 29569896 10.1021/acs.accounts.8b00010

[55] M. Irfan Malik, Z.O. Malaibari, M. Atieh, B. Abussaud, Electrochemical reduction of CO_2_ to methanol over MWCNTs impregnated with Cu_2_O. Chem. Eng. Sci. **152**, 468–477 (2016). 10.1016/j.ces.2016.06.035

[56] J. Bugayong, G.L. Griffin, Electrochemical reduction of CO_2_ using supported Cu_2_O nanoparticles. ECS Trans. **58**, 81–89 (2013). 10.1149/05802.0081ecst

[57] I.V. Chernyshova, P. Somasundaran, S. Ponnurangam, On the origin of the elusive first intermediate of CO_2_ electroreduction. Proc. Natl. Acad. Sci. U.S.A. **115**, E9261–E9270 (2018). 10.1073/pnas.1802256115 30224482 10.1073/pnas.1802256115 PMC6176638

[58] P.-P. Yang, X.-L. Zhang, F.-Y. Gao, Y.-R. Zheng, Z.-Z. Niu et al., Protecting copper oxidation state *via* intermediate confinement for selective CO_2_ electroreduction to C_2+_ fuels. J. Am. Chem. Soc. **142**, 6400–6408 (2020). 10.1021/jacs.0c01699 32176485 10.1021/jacs.0c01699

[59] M. He, C. Li, H. Zhang, X. Chang, J.G. Chen et al., Oxygen induced promotion of electrochemical reduction of CO_2_*via* co-electrolysis. Nat. Commun. **11**, 3844 (2020). 10.1038/s41467-020-17690-8 32737312 10.1038/s41467-020-17690-8 PMC7395777

[60] Q. Li, P. Xu, B. Zhang, H. Tsai, S. Zheng et al., Structure-dependent electrocatalytic properties of Cu_2_O nanocrystals for oxygen reduction reaction. J. Phys. Chem. C **117**, 13872–13878 (2013). 10.1021/jp403655y

