# Supplementary material for: Exploration of Gas-Dependent Self-Adaptive Reconstruction Behavior of Cu2O for Electrochemical CO2 Conversion to Multi-Carbon Products
Source: Nanomicro Lett. 2024 Nov 19;17:66. doi: 10.1007/s40820-024-01568-1 (PMC11573952; doi:10.1007/s40820-024-01568-1)
Supplement: Supplementary file 1 — Supplementary file1 (DOCX 17496 KB) [file 40820_2024_1568_MOESM1_ESM.docx]

Supporting Information for

**Exploration of Gas-Dependent Self-Adaptive Reconstruction Behavior of Cu_2_O for Electrochemical CO_2_ Conversion to Multi-Carbon Products**

Chaoran Zhang^1^, Yichuan Gu^1^, Qu Jiang^1^, Ziyang Sheng^1^, Ruohan Feng^1^, Sihong Wang^1^, Haoyue Zhang^1^, Qianqing Xu^1^, Zijian Yuan^1^, Fang Song^1,^*

^1^State Key Laboratory of Metal Matrix Composites, School of Materials Science and Engineering, Shanghai Jiao Tong University, Shanghai 200240, P. R. China

* Corresponding author. E-mail: [songfang@sjtu.edu.cn](mailto:songfang@sjtu.edu.cn) (Fang Song)

**S1 Supplementary Experimental Section**

**S1.1 Materials Characterizations**

The crystallographic information of Cu_2_O nanocrystals was collected by Mini Flex 600 with Cu Kα irradiation (λ = 1.5406 Å) at 40 kV and 40 mA in the 2θ range of 5 to 80° with a scanning rate of 10°/min. TESCAN MIRA3 field emission scanning electron microscopy (FE-SEM) equipped with energy dispersive spectroscopy (EDS) was applied to confirm the morphology and elements of the products. Transmission electron microscopy (TEM) and high-resolution Transmission electron microscopy (HRTEM) were collected by Talos F200X G2 FETEM under an acceleration voltage of 200 kV. Electron energy-loss spectra (EELS) were fulfilled via Thermo Scientific Spectra 300 (S)TEM equipped with aberration corrector. X-ray photoelectron spectroscopy (XPS) analysis was obtained by AXIS UltraDLD and all spectra were corrected using the C 1s line at 284.6 eV. Raman spectra were performed on a confocal Raman microscope with a laser wavelength of 532 nm (Renishaw inVia Qontor 27).

**S1.2 Electrochemical Measurements**

All electrochemical measurements were carried out on a Gamry Reference 3000 electrochemical instrument. The automatic iR compensation (85%) was used. Ag/AgCl electrode with a 3.5M KCl filling solution was used as the reference electrode. Potential versus RHE was calculated as E_vs.RHE_ = E_vs.Ag/AgCl_ + 0.2046 V + 0.0592 V × pH. The pH values of the electrolytes were measured by a pH meter (FE28 Standard, Mettler Toledo). The pH values of CO_2_ and N_2_ saturated 0.5 M KHCO_3_ electrolytes used in this work are 7.25 and 8.36, respectively.

A carbon paper with a gas diffusion layer (Sigracet 28BC, SGL) was used as the working electrode for electrolysis at a constant potential. The area exposed to the electrolyte was fixed at 1 cm^2^. 3 mg of catalyst and 40 μL of a Nafion^®^ perfluorinated resin solution were dispersed in 1 mL mixed solution of ethanol and water with a volume ratio of 1:1. The mixture was treated with ultrasound for 30 min and 100 μL of the mixture was dropped on the carbon paper. The electrode was dried naturally for 1 hour. The loading on the electrode was 0.3 mg cm ^-2^.

An H-type cell made of glass was used. Working and reference electrodes were fixed in one chamber and the counter electrode was fixed in the other chamber. The two chambers were separated by an anion exchange membrane (Fumapem FAA-3-50). A bubble sieve was fixed at the gas inlet to generate small bubbles. A Pt plate was used as the counter electrode. 0.5 M KHCO_3_ electrolyte was used for both chambers.

A flow cell system was assembled using the catalysts as working electrodes, Ag/AgCl (3.5 M KCl-filled) electrode as reference electrode, and nickel foam as counter electrode. The flow cell electrolyzer comprises polyetheretherketone (PEEK) and a silicone gasket for sealing. The gas flow rate was fixed at 10 s.c.c.m. using a mass-flow controller (Sevenstar CS-200A). The CO_2_ gas flows from the back of the carbon paper to the catalyst side to participate in the reaction. The catholyte and anolyte were 1M KOH and separated by a Sustainion^®^ X37-50 film (Grade RT), the flow rate was adjusted to 10 mL min^-1^ by a peristaltic pump.

**S1.3 Products Analysis**

Gas products were detected and quantified via gas chromatography (Trace 1310, ThermoFisher Scientific, USA), including CO, CH_4_, C_2_H_4_, C_2_H_6_ and H_2_. Three GC columns (Porapak N, TG-BOND Q+, and Molesieve 5A) are used to separate different gases.

Faradic efficiency (FE) can be obtained with:

$$FE=\frac{nzF}{Q}=\frac{x_{i}pV_{0}zF}{RT\int Idt}$$

liquid products were analyzed and quantified by a ^1^H nuclear resonance (NMR) spectroscopy.

Faradic efficiency (FE) can be obtained with:

$$FE=\frac{nzF}{Q}=\frac{CV_{L}zF}{\int Idt}$$

where n is the moles of generated products, z is the number of transferred electrons for each product, F is the Faradaic Constant of 96485 C mol^-1^, Q is the total charge during the reaction, x_i_ is the fraction of products detected by GC, p is the ambient pressure, V_0_ is the analyzed gas volume in GC, T is the ambient temperature, ∫Idt is the integrated charge, V_L_ is the catholyte volume, C is the molar concentration of liquid products in the sampling solution.

**S2 Supplementary Figures and Tables**


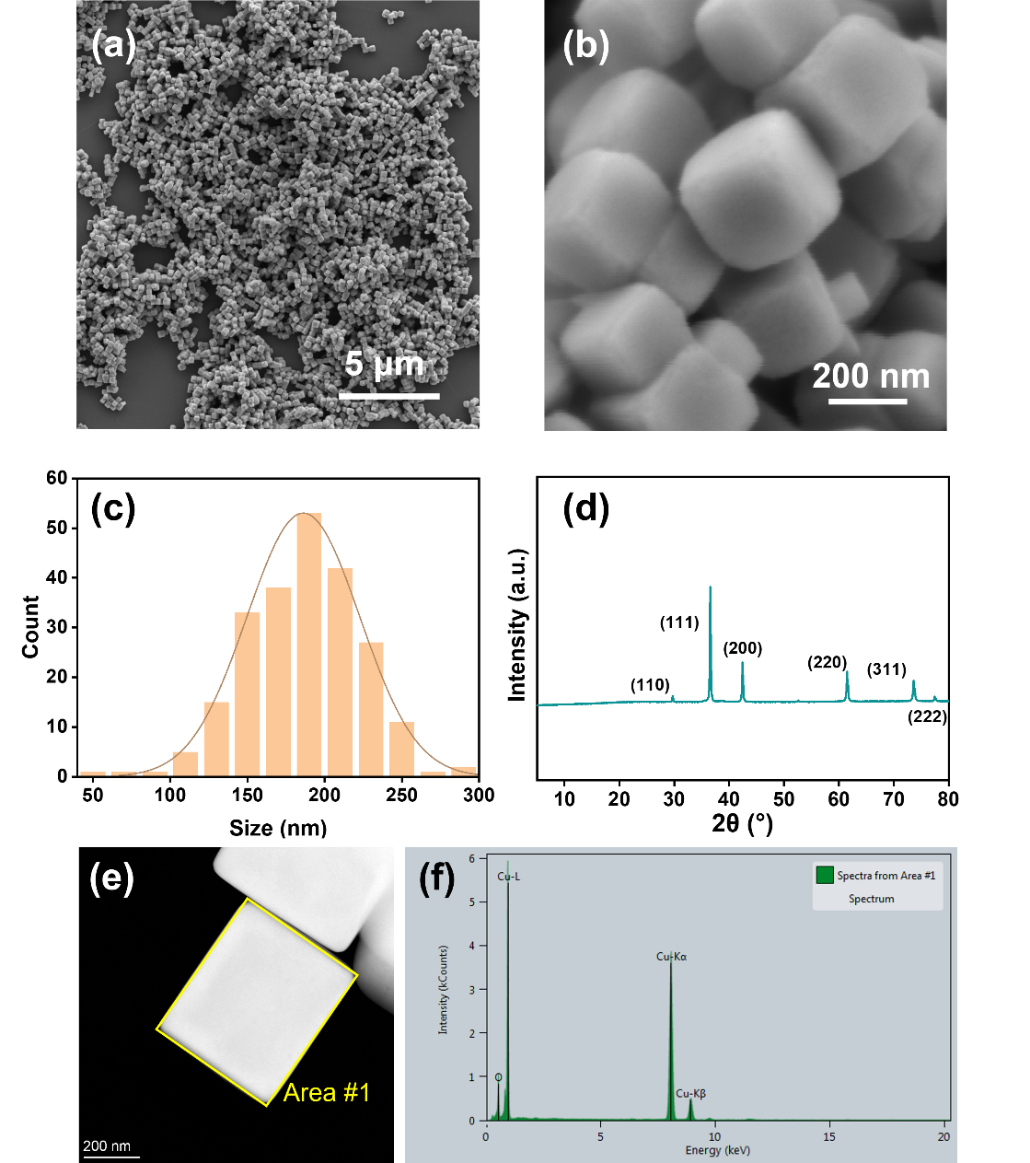


**Fig. S1** **Microstructures and size distribution of cubic Cu_2_O nanocrystals.** **a**) and **b**) SEM images; **c**) size distribution and **d**) XRD pattern of Cu_2_O cube; **e**) HADDF-STEM image of Cu_2_O nanocube; f) Elemental distribution of area #1 in e)


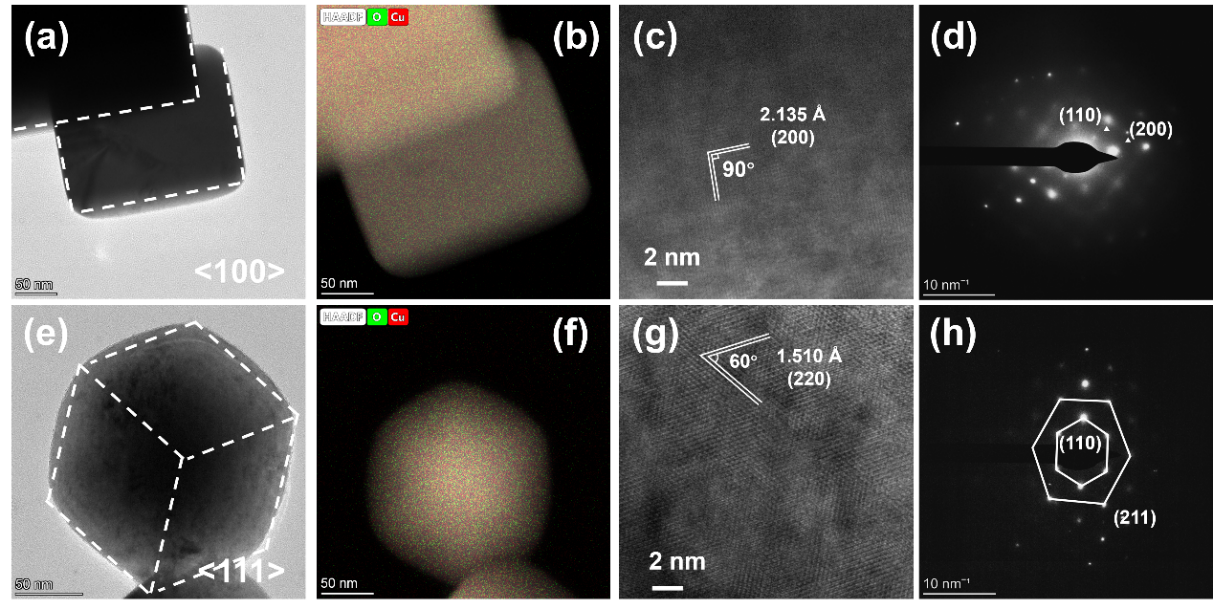


**Fig. S2 TEM images of Cu_2_O nanocubes.** **a-d**) Observation with electron beams parallel to [100], **e-h**) Observation with electron beams parallel to [111]


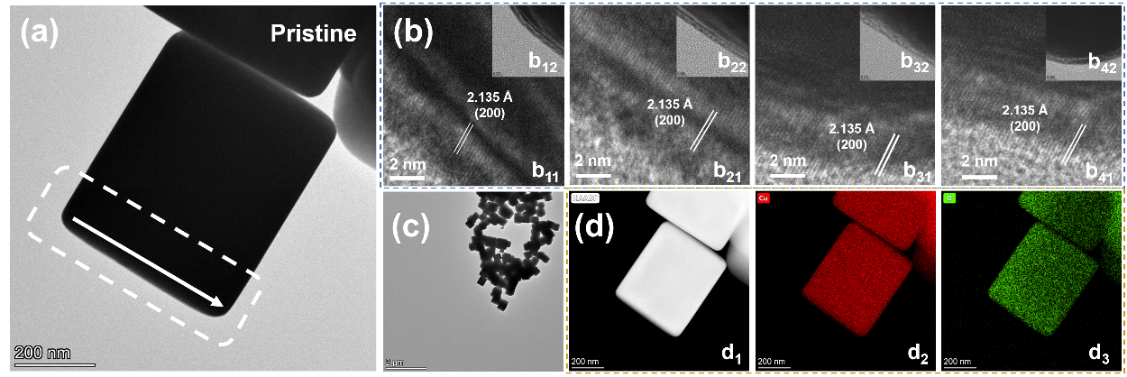


**Fig. S3 TEM images of pristine Cu_2_O nanocubes.** **a**) microstructure under bright-field; **b**) HRTEM images along the arrow direction in a; **c**) low-magnification TEM images; **d**) HADDF image and elemental mapping. HRTEM: b_11_-b_41_) enlarged area of the inset parts which are selected along the arrow direction in the dash line-enclosed area of a), b_12_-b_42_) the selected parts in the dash line-enclosed area of a)


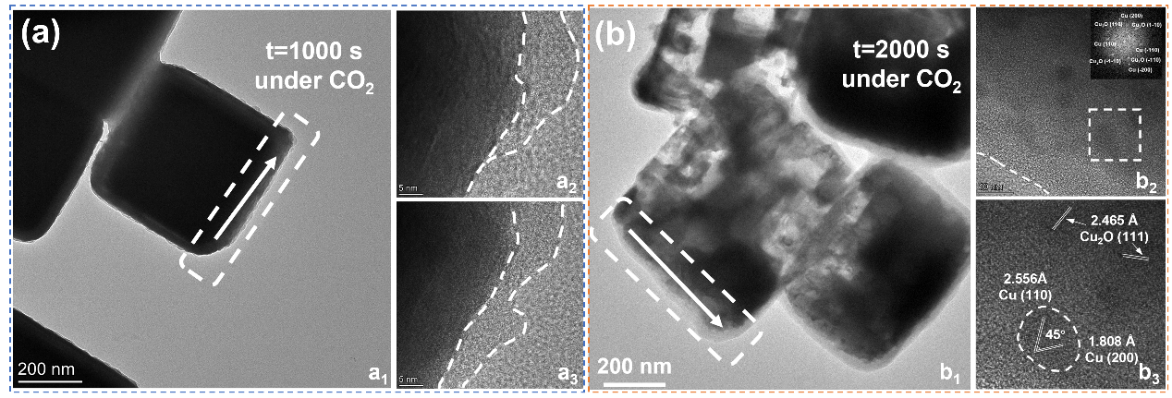


**Fig. S4 TEM images of Cu_2_O Cube after activation in CO_2_.** **a**) microstructure and HRTEM images along the arrow direction after 1000s-activation, **b**) HRTEM images after 2000s-activation, and the supplementary demonstration of Cu(0)@Cu_x_O structure. HRTEM: a_2_-a_3_) enlarged dash-lined enclosed area in a_1_), two dash curves are borders between crystalline Cu_2_O and amorphous Cu_x_O; b_2_) enlarged dash-lined enclosed area in b_1_); b_3_) enlarged dash-lined enclosed area in b_2_). SAED: inset of b_2_) is the SAED pattern of b_2_)


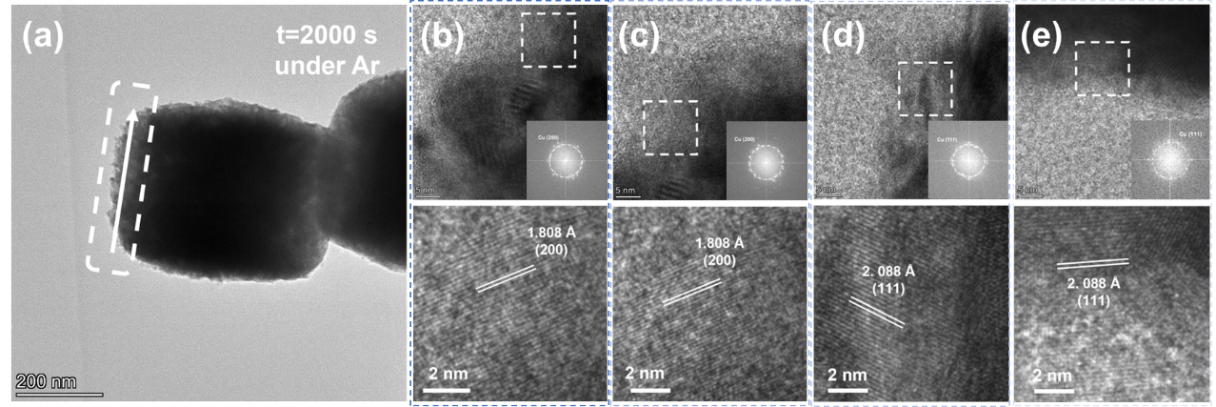


**Fig. S5 TEM images of Cu_2_O Cube after activation in Ar.** **a**) microstructure of Cu_2_O Cube after 2000s-activation in Ar, **b-e**) HRTEM images along the arrow direction after 2000s-activation. HRTEM: b-e) enlarged area of the inset parts which are selected along the arrow direction in the dash line-enclosed area of a); SAED: insets of b-e) are the SAED patterns of corresponding ones


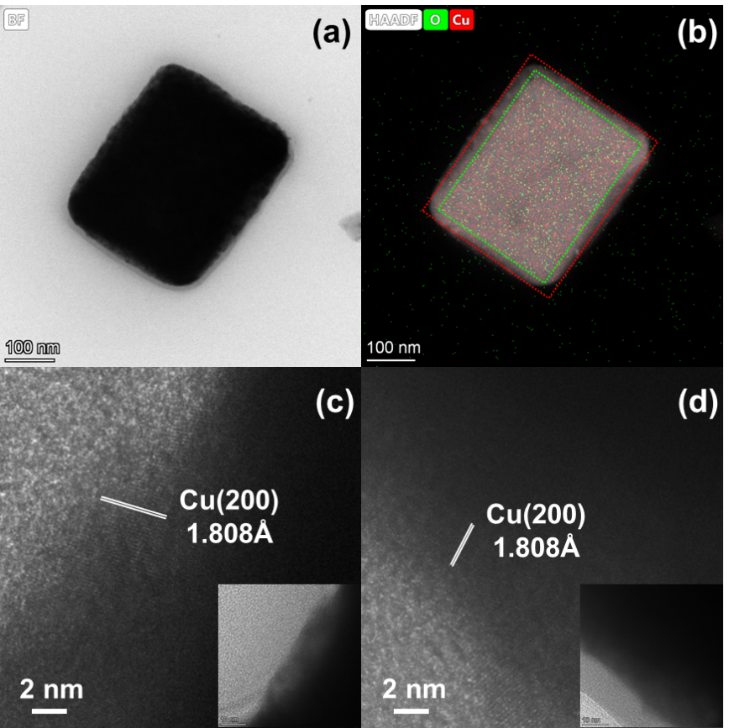


**Fig. S6** Supplementary TEM information of Cu_2_O-Ar. **a**) TEM image, **b**) HADDF and EDS mapping, **c-d**) HRTEM image of Cu_2_O-Ar, insets are corresponding local area of Cu_2_O-Ar surface


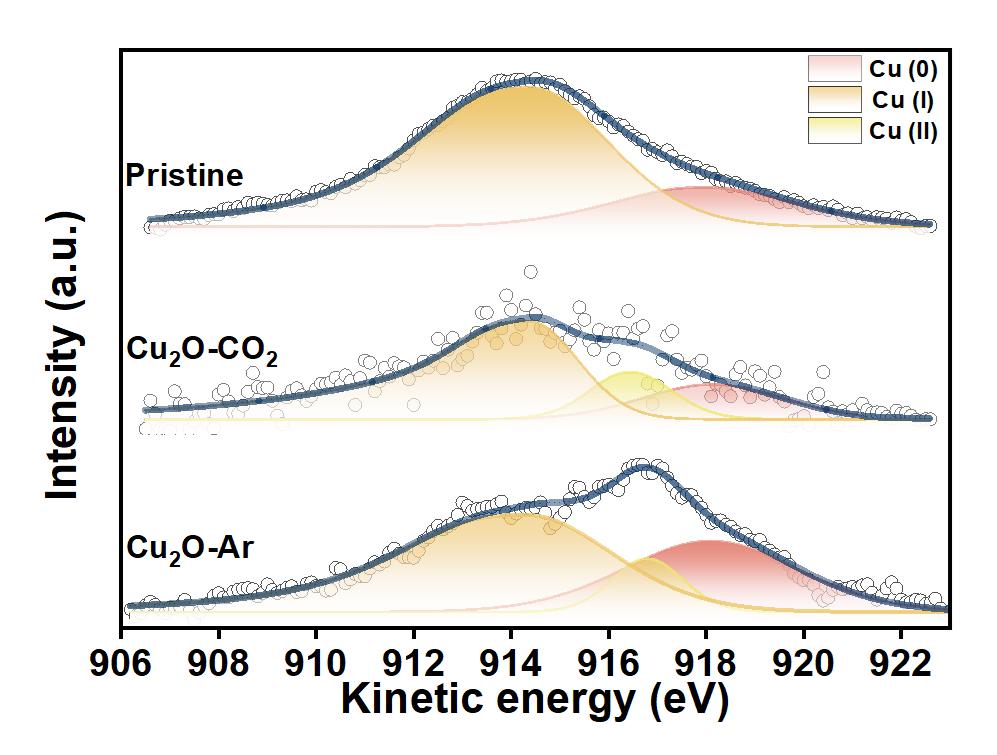


**Fig. S7** Cu LMM Auger spectra of pristine Cu_2_O and those after pre-electrolysis in CO_2_ or Ar atmospheres for 2000 s


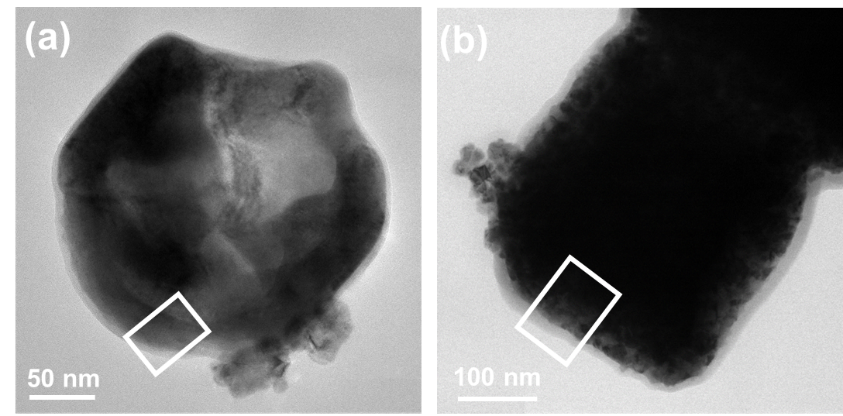


**Fig. S8** TEM images of a selected area in Fig. 2. **a**) Cu_2_O-CO_2_ and **b**) Cu_2_O-Ar


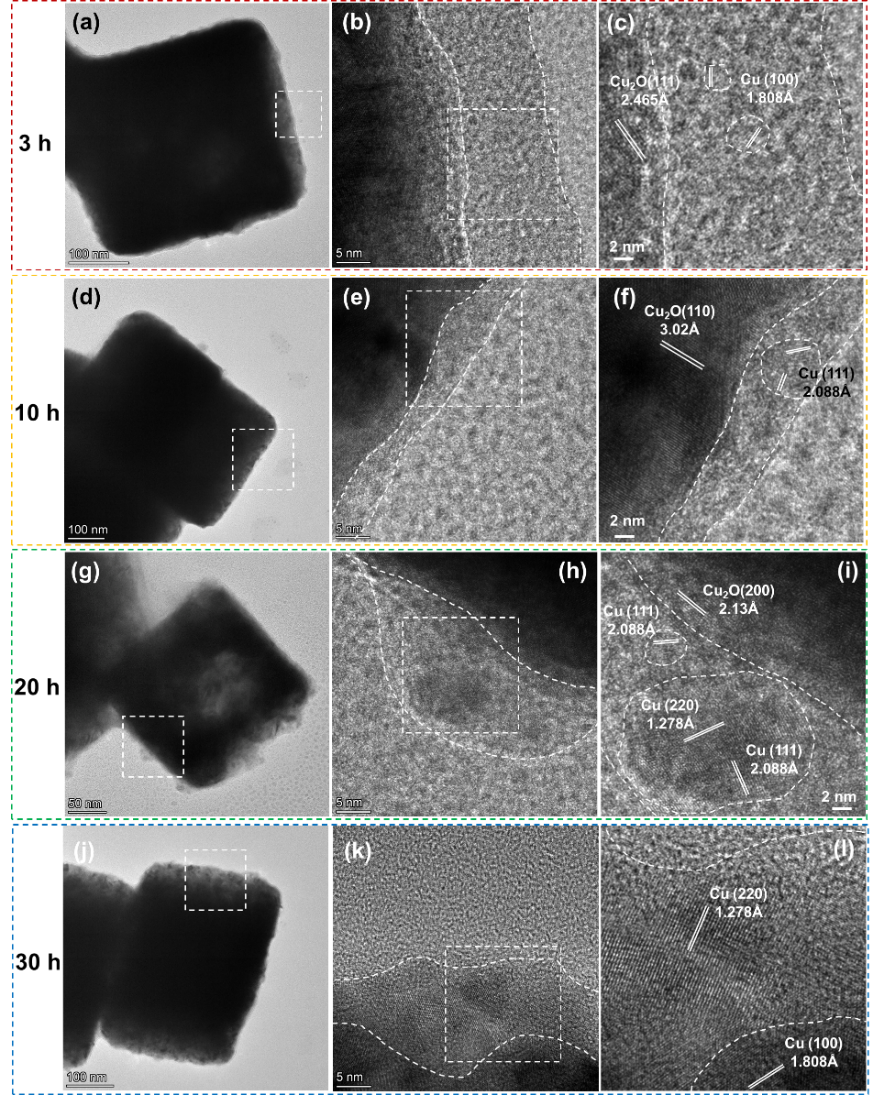


**Fig. S9 Structural evolution of Cu_2_O nanocubes during the electrolysis at a potential of -1.1 V vs. RHE in an H-cell.** TEM and HRTEM for Cu_2_O-CO_2_ at **a-c**) 3 h, **d-e**) 10 h, **g-i**) 20 h, and **j-l**) 30 h


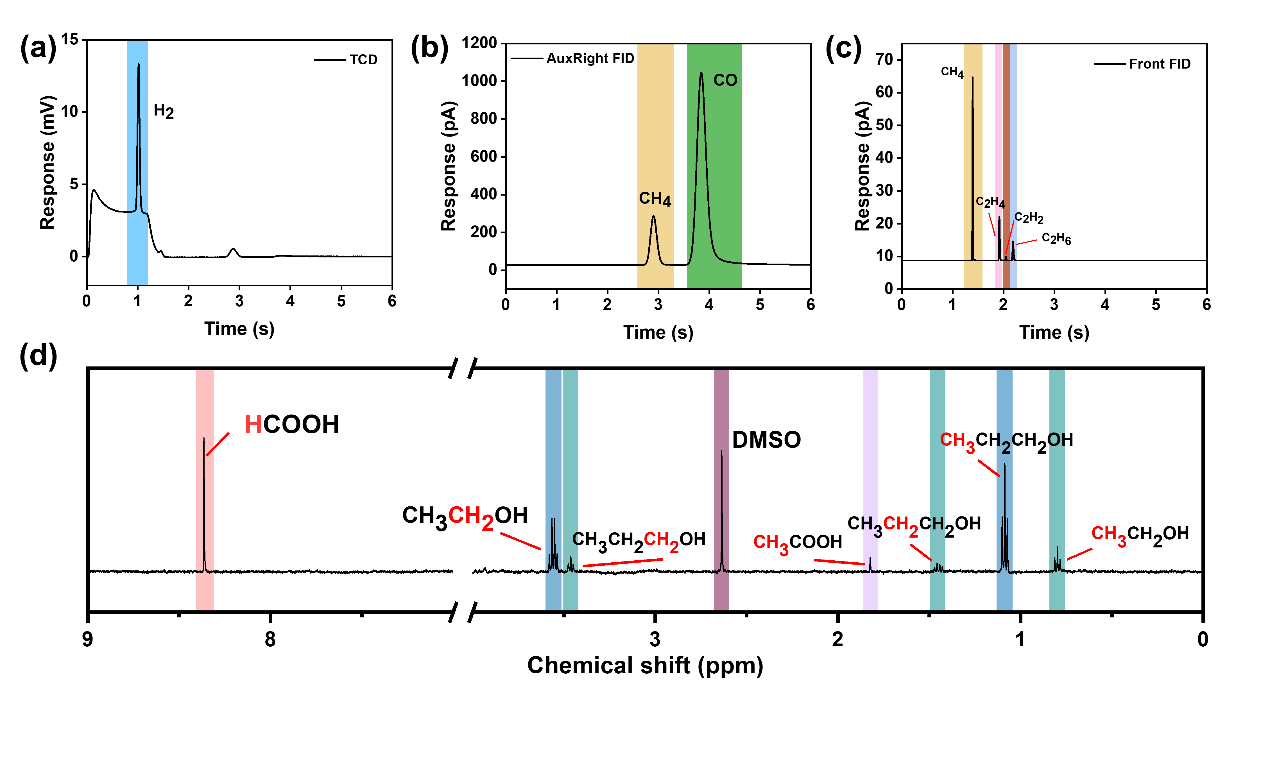


**Fig. S10. Typical product analysis.** (**a**)TCD detector for H_2_; (**b**)Aux-right FID for CH_4_ and CO; (**c**) Front FID detector for gaseous hydrocarbon and (**d**) ^1^H NMR spectroscopy for liquid products


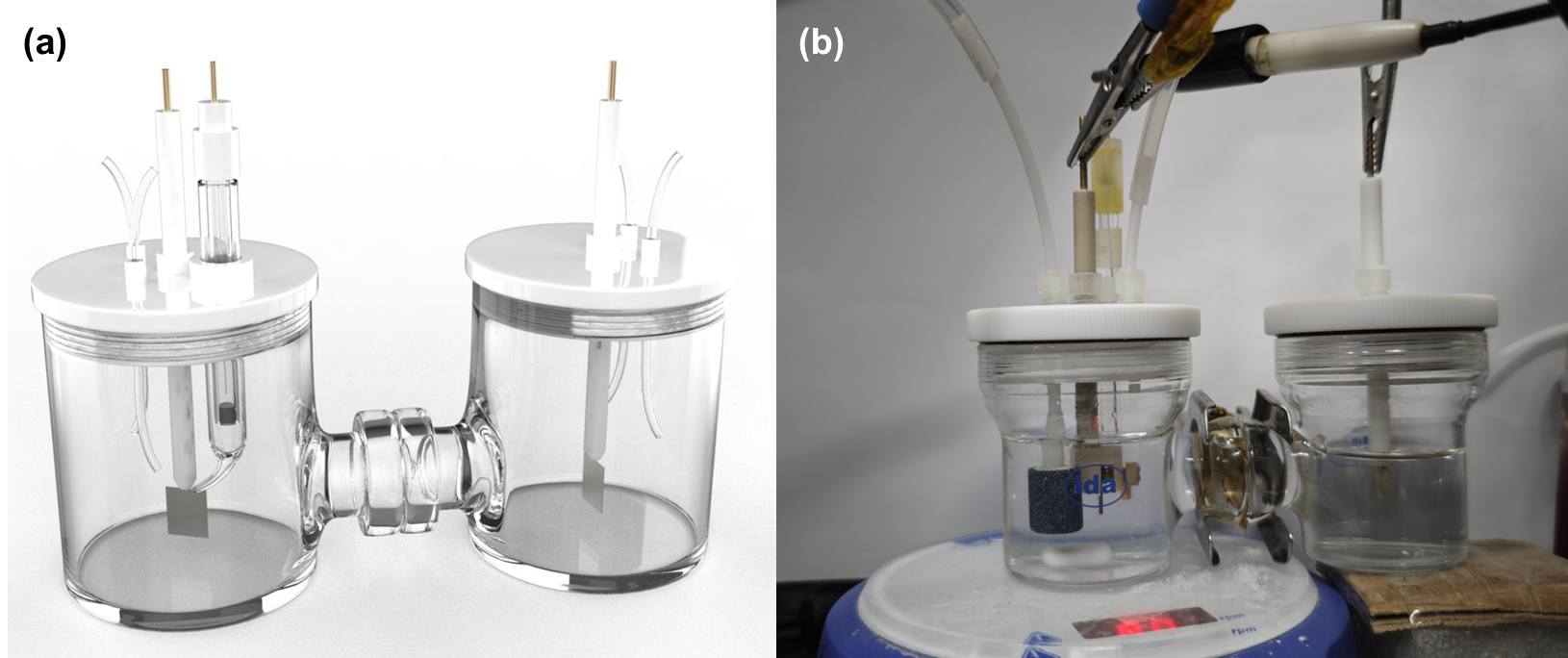


**Fig. S11** Schematic graph (**a**) and digital photograph (**b**) of H-cell


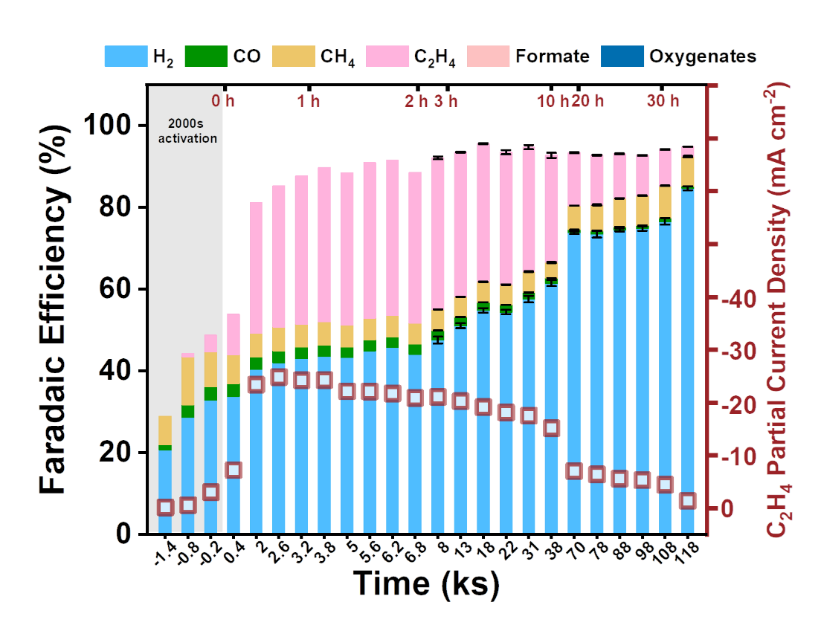


**Fig. S12** Partial current density and Faradaic efficiency of main CO_2_RR products after activation in CO_2_ including activation process for Cu_2_O Cube. Three columns highlighted by the light-grey area indicate the activation process


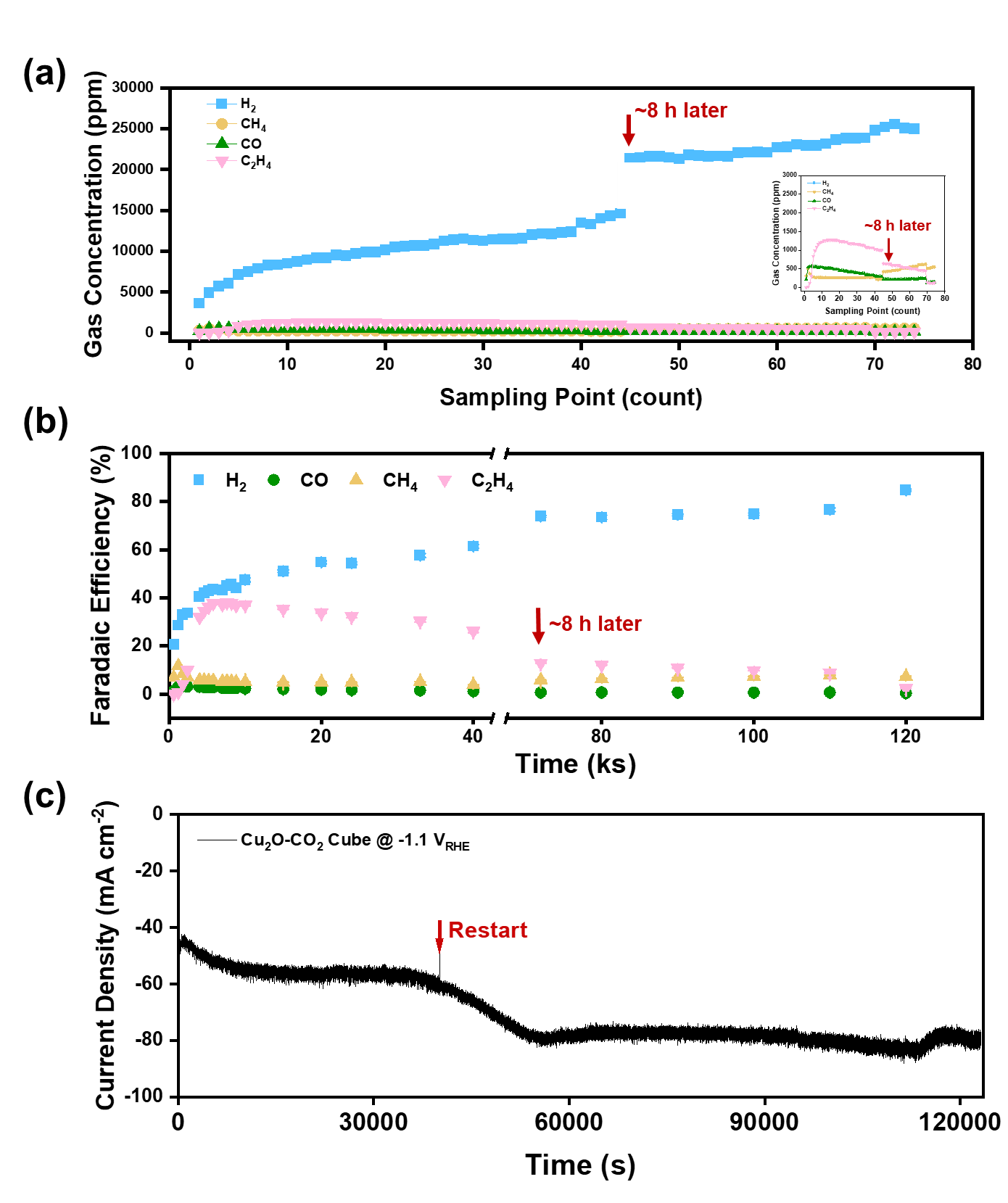


**Fig. S13** **Sampling information and current density of Cu_2_O Cube activated in CO_2_. a**) Sampling information of all products; **b**) FE as a function of time; **c**) Current density in the continuous electrolysis at -1.1 V vs. RHE. The inset of a) is sampling information of CRR products. The discontinuous mutations in a-c) correspond to a period of 8.9 h when we retained the electrolysis but did not collect the data (during the night)


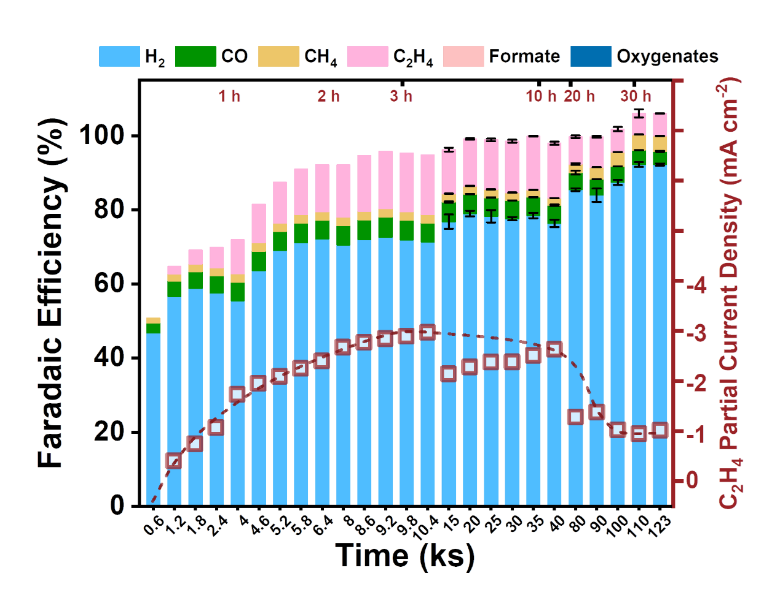


**Fig. S14** Enlarged demonstration of partial current density with Faradaic efficiency of main CO_2_RR products after activation in Ar for Cu_2_O Cube. Red hollow squares are C_2_H_4_ partial current densities at each sampling time


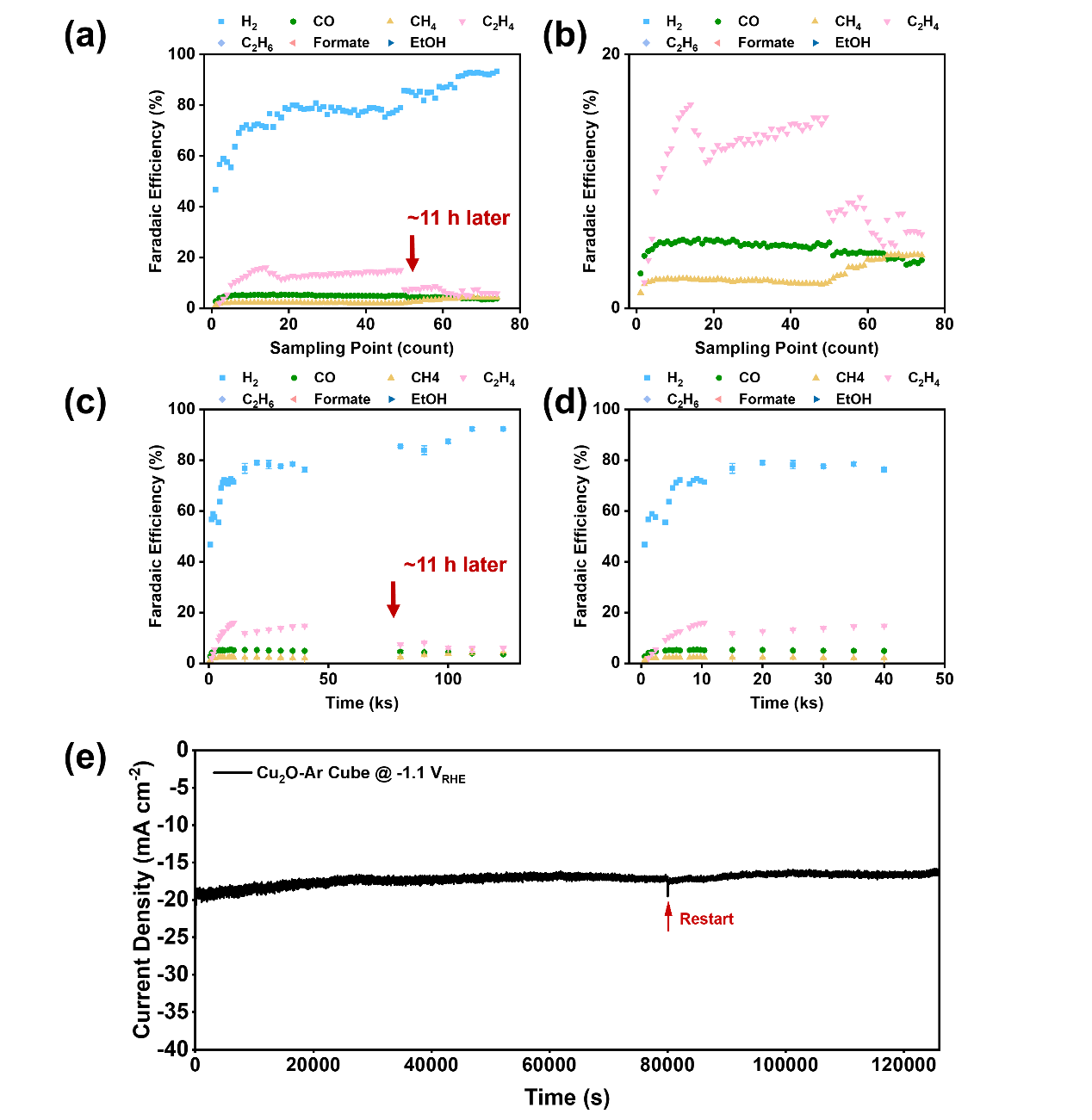


**Fig. S15 Sampling information and current density of Cu_2_O Cube activated in Ar.** **a-b**) sampling detail of products, **c-d**) the FE trends as a function of time, **e**) current density of continuous test at -1.1 V vs. RHE. b) is the lower part of a) to demonstrate the concentrations of CO, CH_4,_ and C_2_H_4_; d) is the earlier part before 40000 s of c) to demonstrate the FEs of CO, CH_4,_ and C_2_H_4_


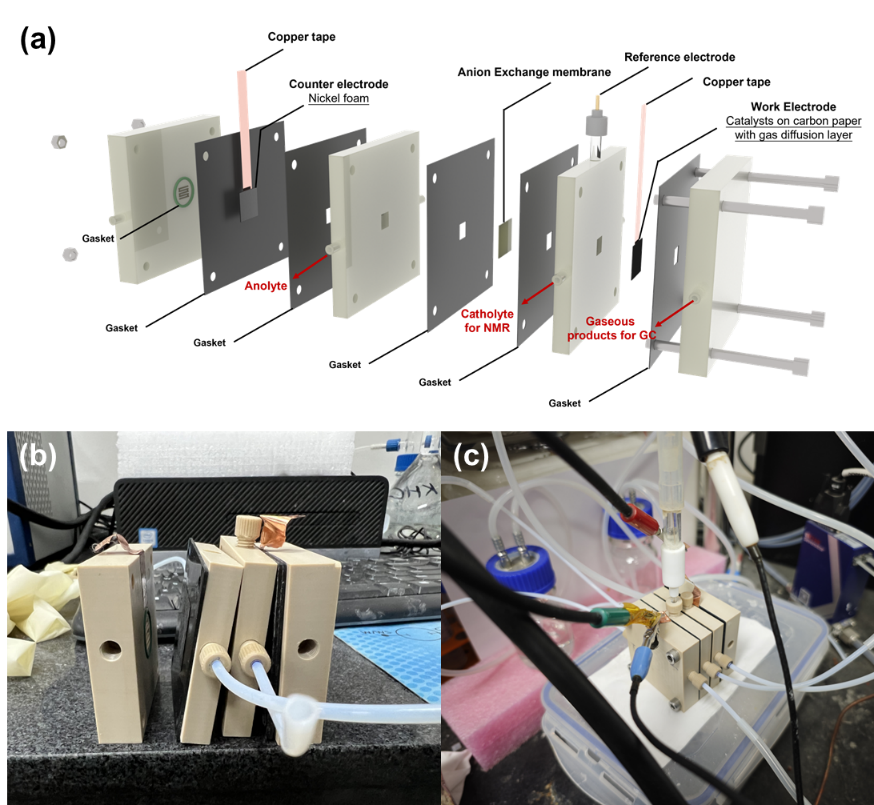


**Fig. S16** Schematic graph (**a**) and digital photographs (**b-c**) of the flow cell


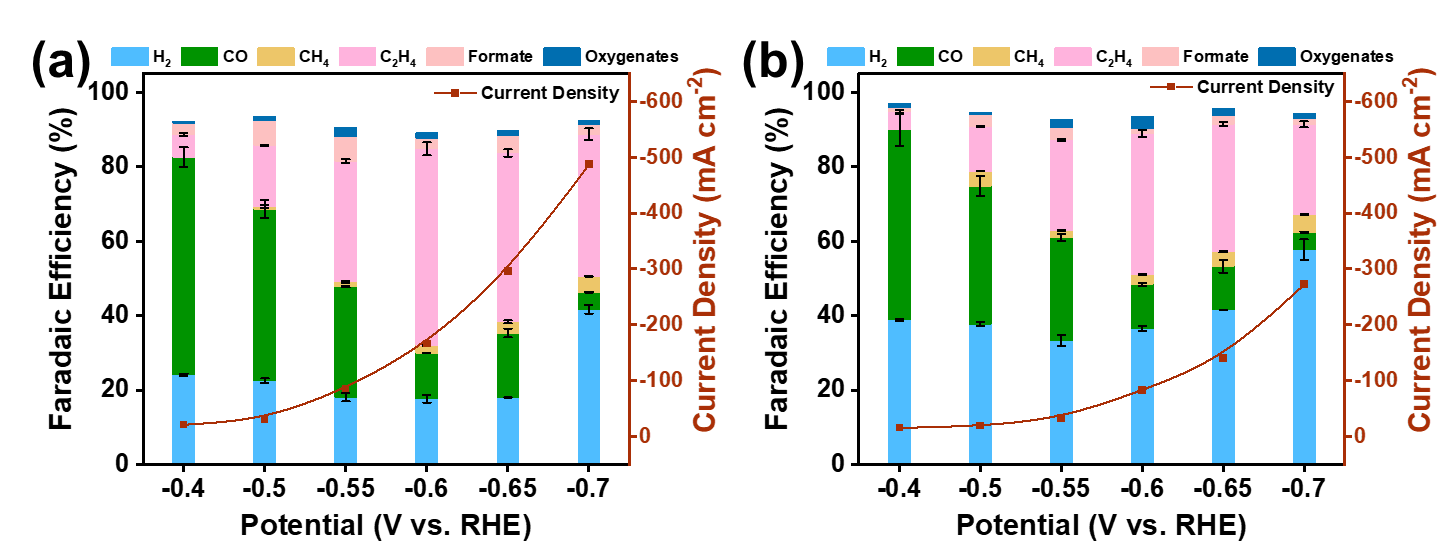


**Fig. S17** Total current density and Faradaic efficiency of main CO_2_RR products after activation in CO_2_. a) activation in 0.5 M KHCO_3_ and evaluation in 1 M KOH, b) activation and evaluation in KHCO_3_


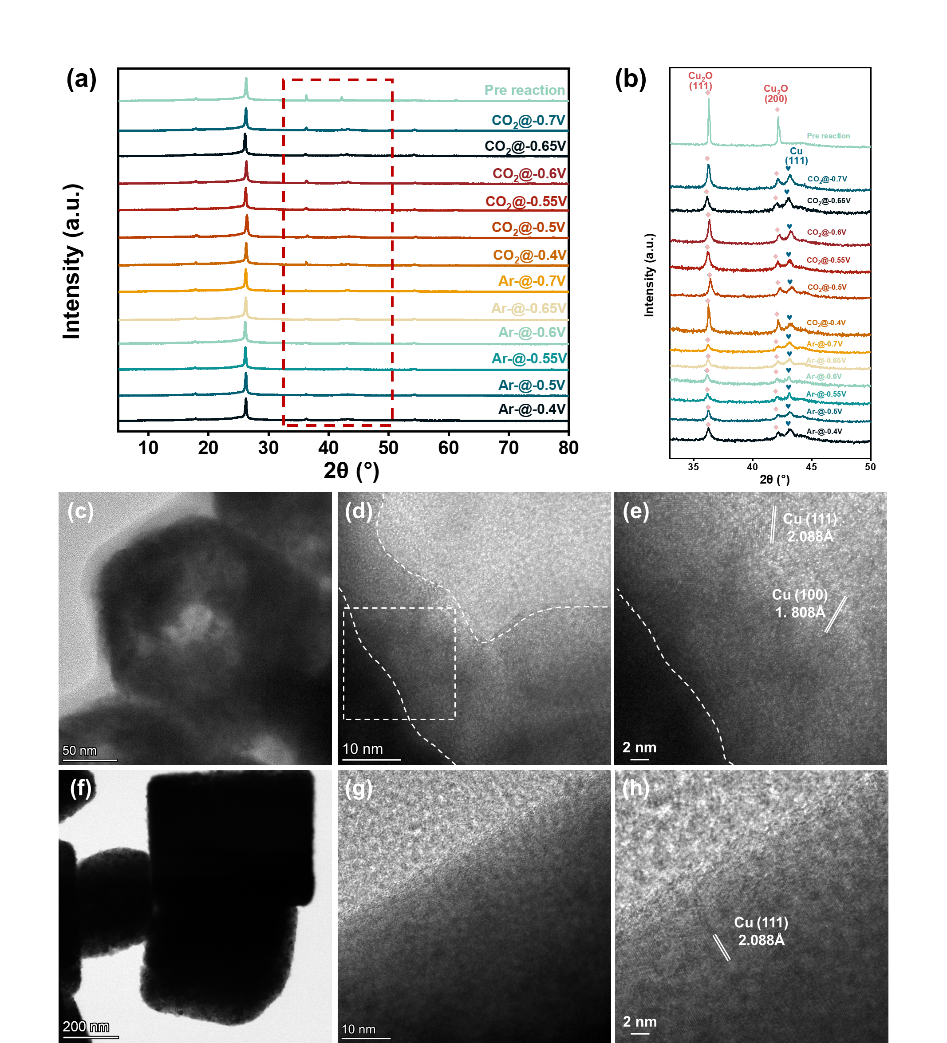


**Fig. S18 Microstructure of Cu_2_O after activation in the flow cell.** XRD patterns as a function of activation potentials in CO_2_ and Ar. **a**) full spectra information and **b**) XRD patterns at a selected range (red dash line-enclosed area of a) of 2θ from 30° to 50°. Structural evolution of Cu_2_O nanocubes in the pre-electrolysis at a potential of -0.7 V vs. RHE in a flow cell. TEM and HRTEM images of Cu_2_O after activation in **c-e**) CO_2_ and **f-h**) Ar


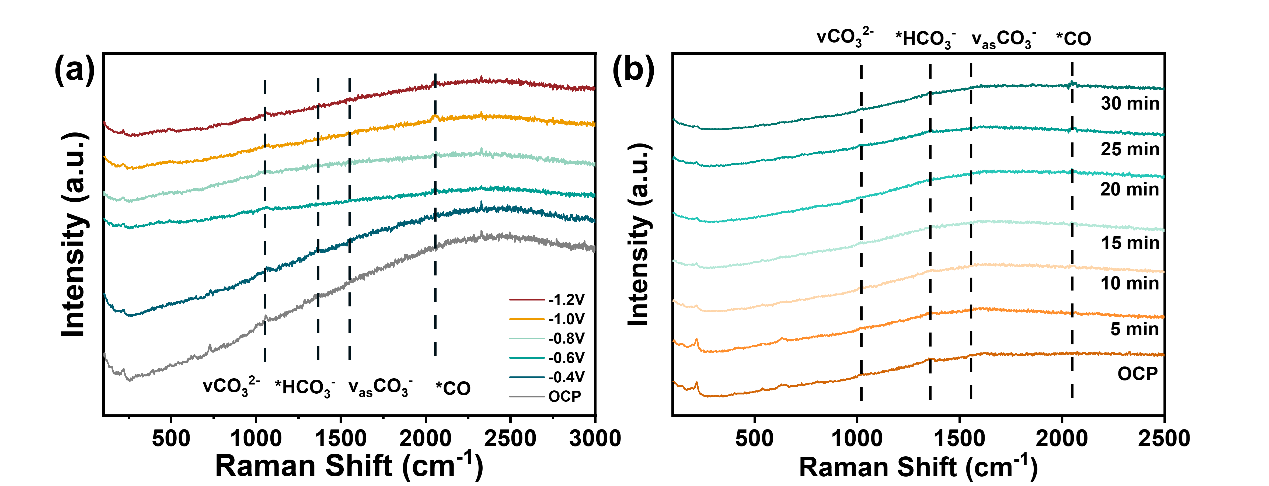


**Fig. S19 Full range spectra of *in situ* Raman spectra of Cu_2_O-CO_2_.** **a**) potential-dependent spectra and **b**) time-dependent spectra. Black vertical dash lines are the intermediate signals labeled


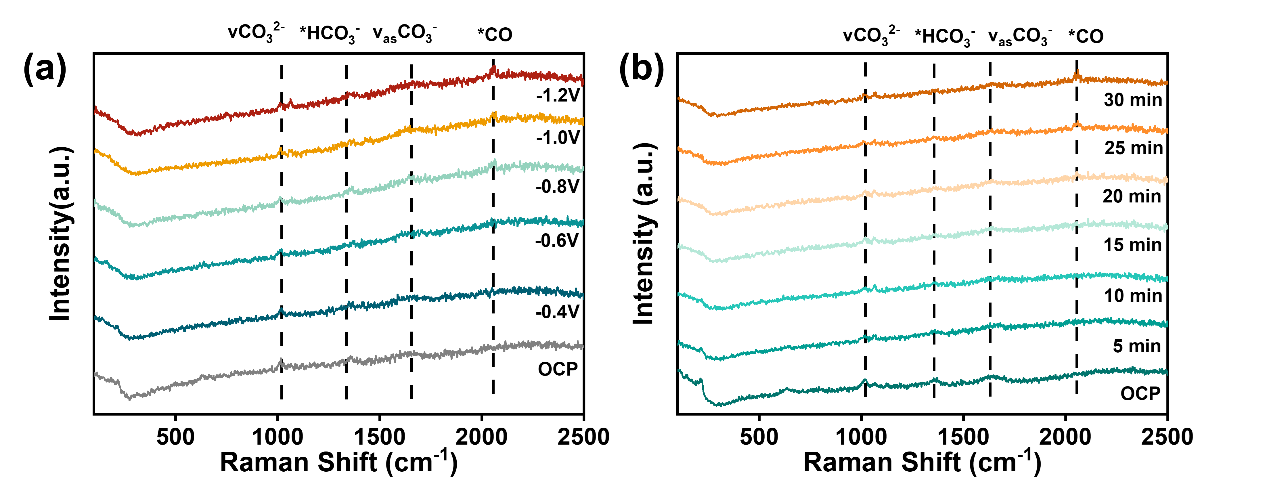


**Fig. S20 Full range spectra of *in situ* Raman spectra of Cu_2_O-Ar.** **a**) potential-dependent spectra and **b**) time-dependent spectra. Black vertical dash lines are the intermediate signals labeled


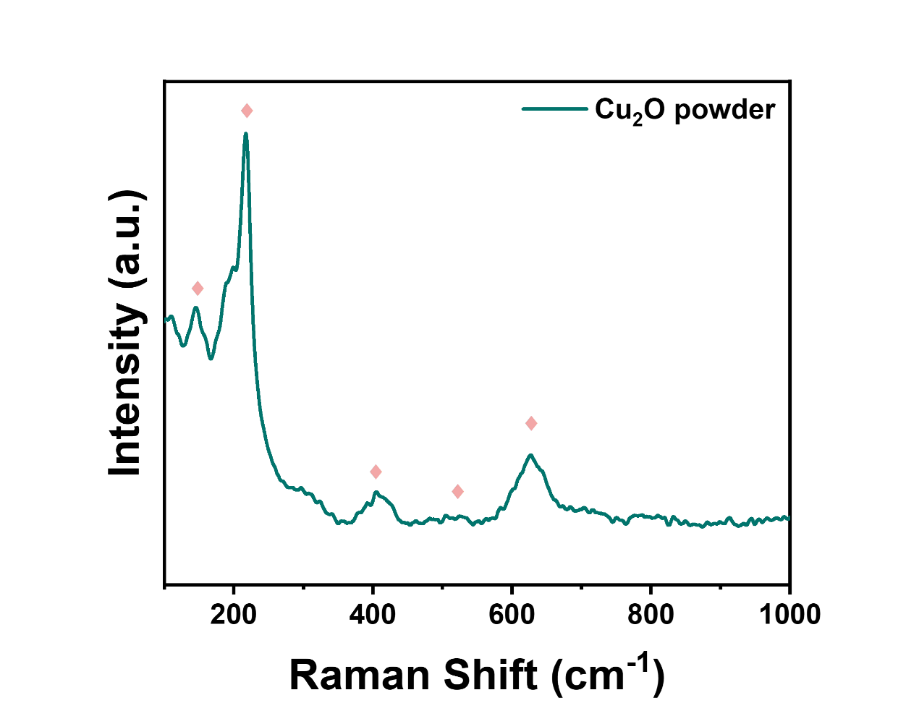


**Fig. S21** Raman spectra of Cu_2_O powders. The typical peaks of Cu_2_O are labeled with pink rhombus


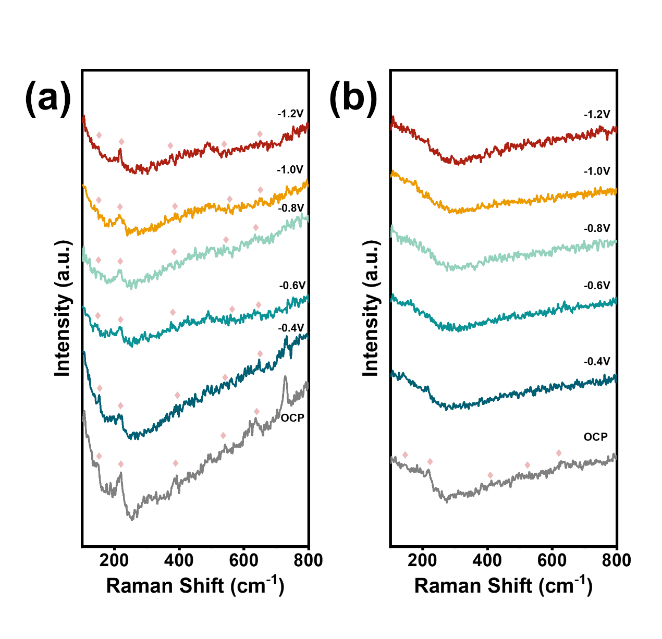


**Fig. S22 The characteristic Raman peaks of Cu_2_O as a function of potentials.** a) Cu_2_O-CO_2_ and b) Cu_2_O-Ar. The typical peaks of Cu_2_O are labeled with pink rhombus


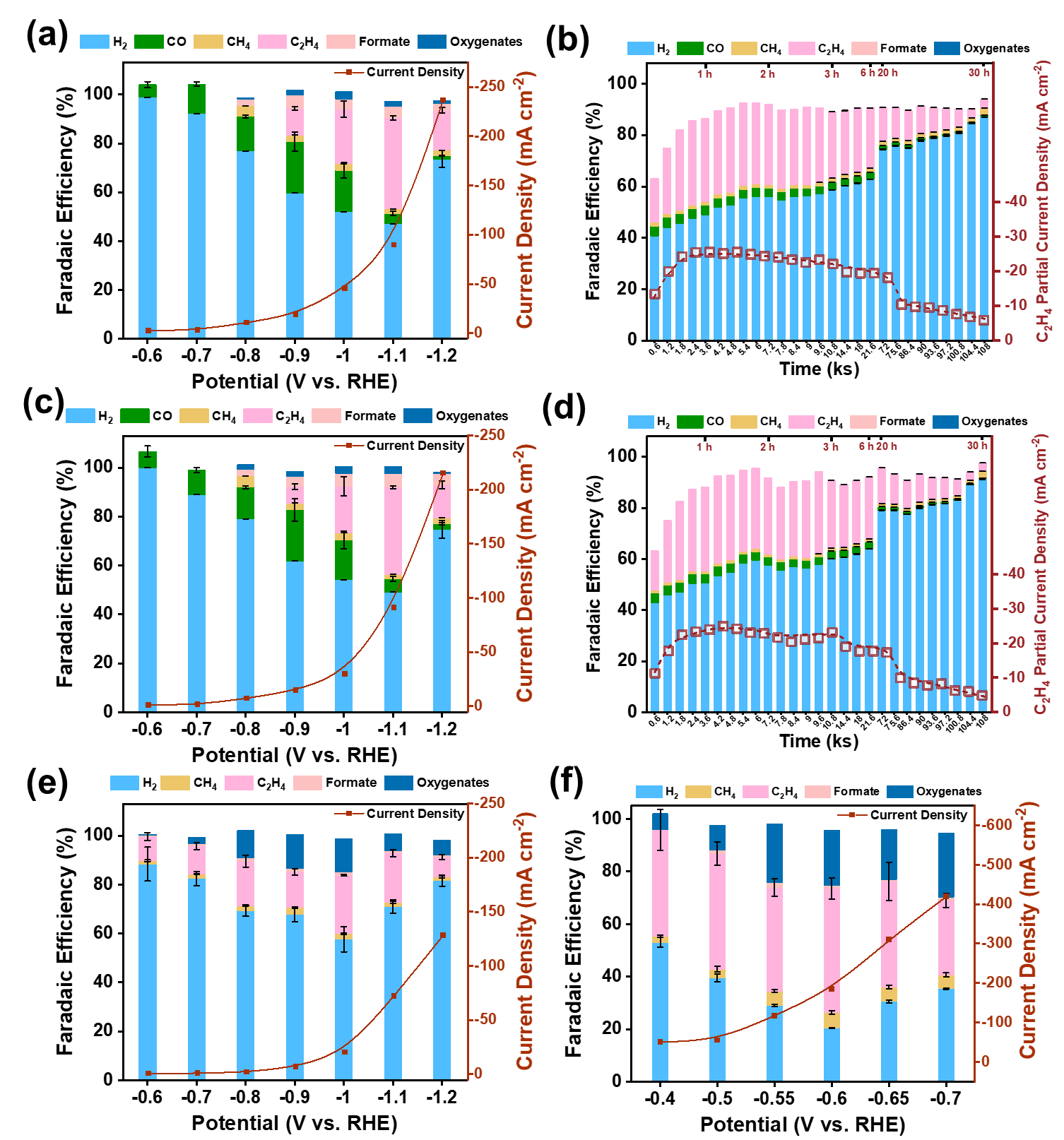


**Fig. S23 Current density and Faradaic efficiency of main CO_(2)_RR products after activation in different atmospheres**. **a-b**) under pure CO atmosphere; **c-d**) under CO_2_ and CO mixed atmosphere (volume ratio of CO_2_ to CO is 1:1); **e-f**) CORR products of Cu_2_O-CO_2_ under an H-cell and a flow cell configuration


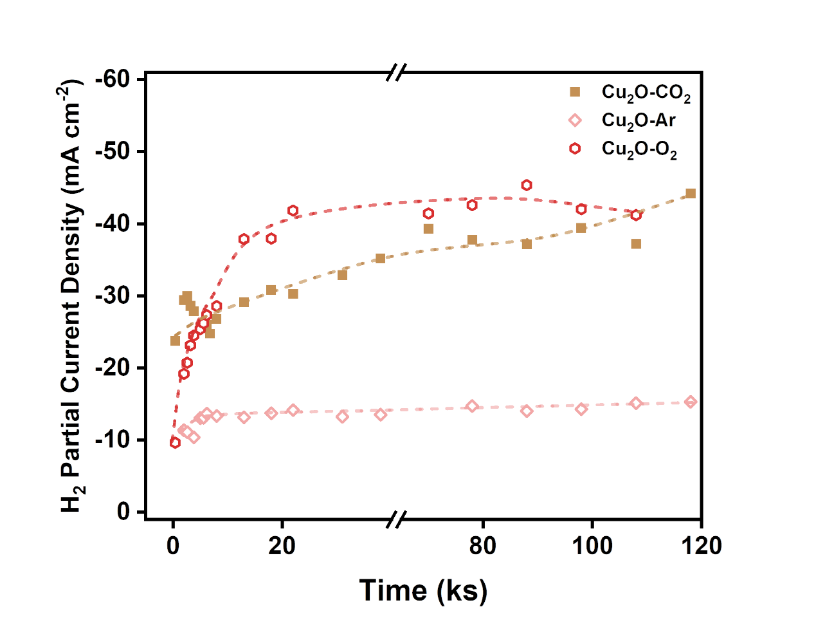


**Fig. S24** H_2_ Partial current density of Cu_2_O-CO_2_, Cu_2_O-Ar and Cu_2_O-O_2_


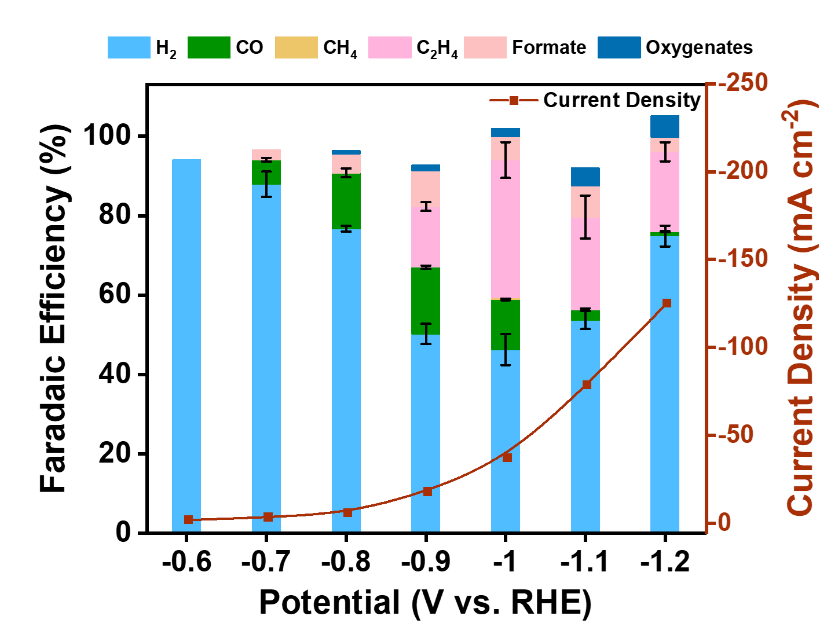


**Fig. S25** Total current density and Faradaic efficiency of the main CO_2_RR products after activation in O_2_ for Cu_2_O Cube


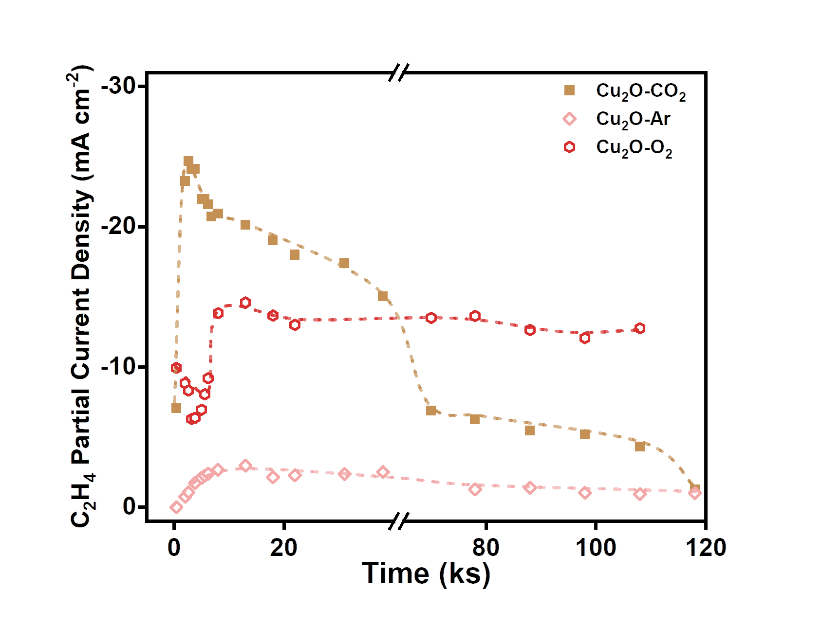


**Fig. S26** The dependence of partial current density of C_2_H_4_ on different gas feeding activations


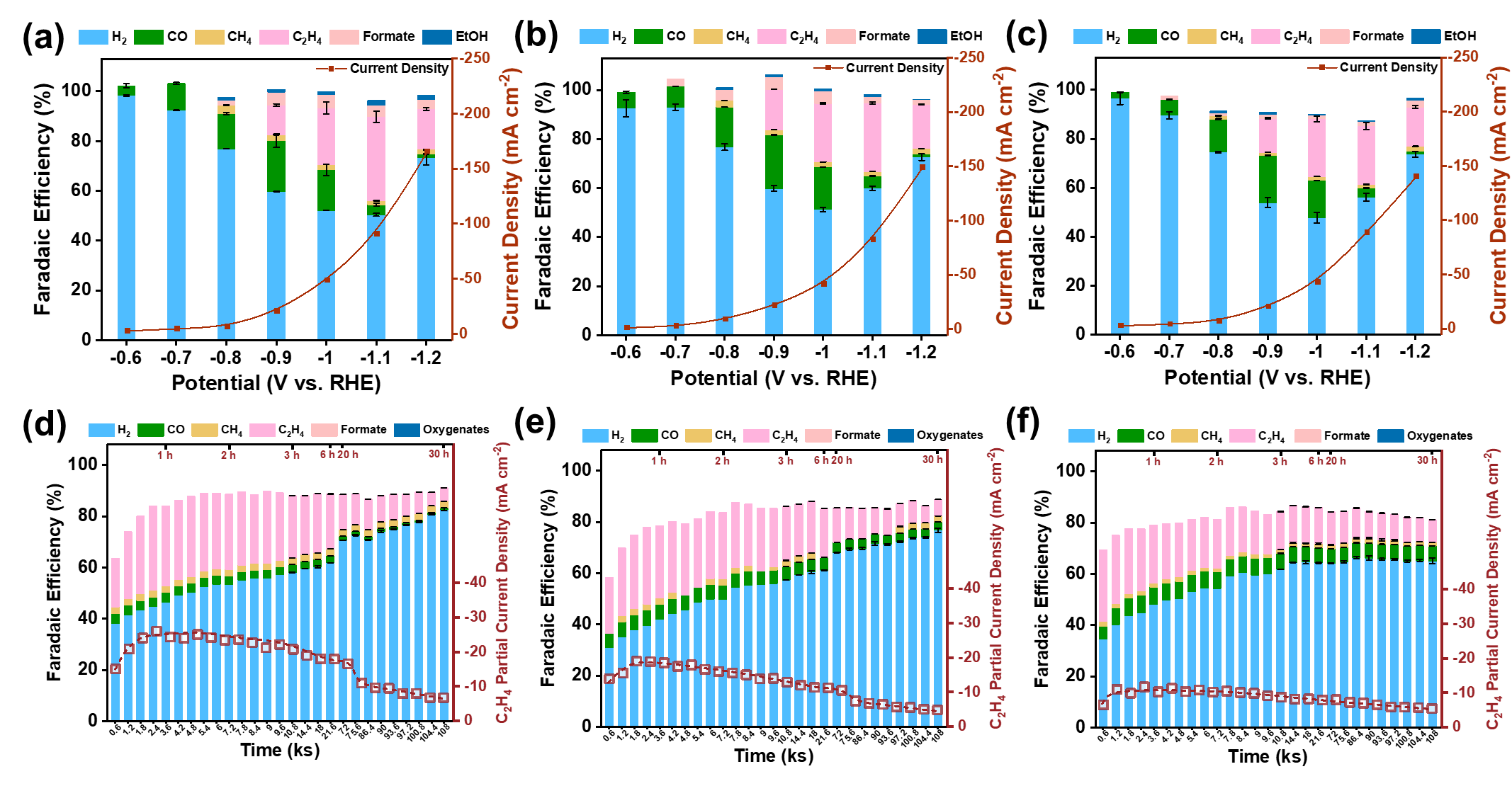


**Fig. S27 Partial current density and Faradaic efficiency of main CO_2_RR products after activation in mixed CO_2_ and O_2_**. **a** and **d**) volume ratio of CO_2_ and O_2_ equals 3:1, **b** and **e**) volume ratio of CO_2_ and O_2_ equals 2:2, **c** and **f**) volume ratio of CO_2_ and O_2_ equals 1:3


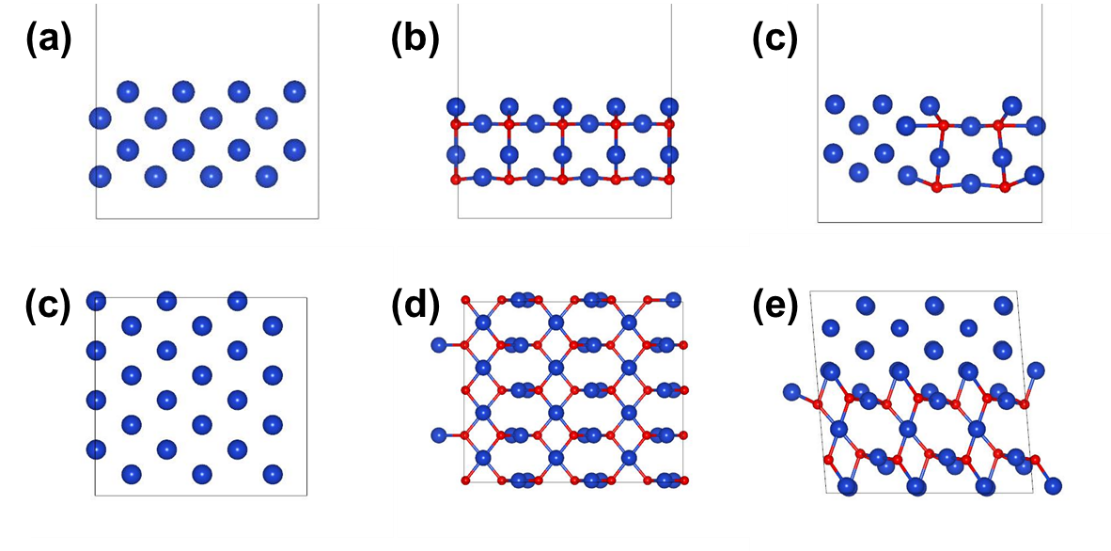


**Fig. S28 Optimized geometry of Cu(110), Cu_2_O(110), and Cu/Cu_2_O.** Top view of **a**) Cu(110), **b**) Cu_2_O(110) and **c**) Cu/Cu_2_O interface; side view of **c**) Cu(110), **d**) Cu_2_O(110) and **e**) Cu/Cu_2_O interface


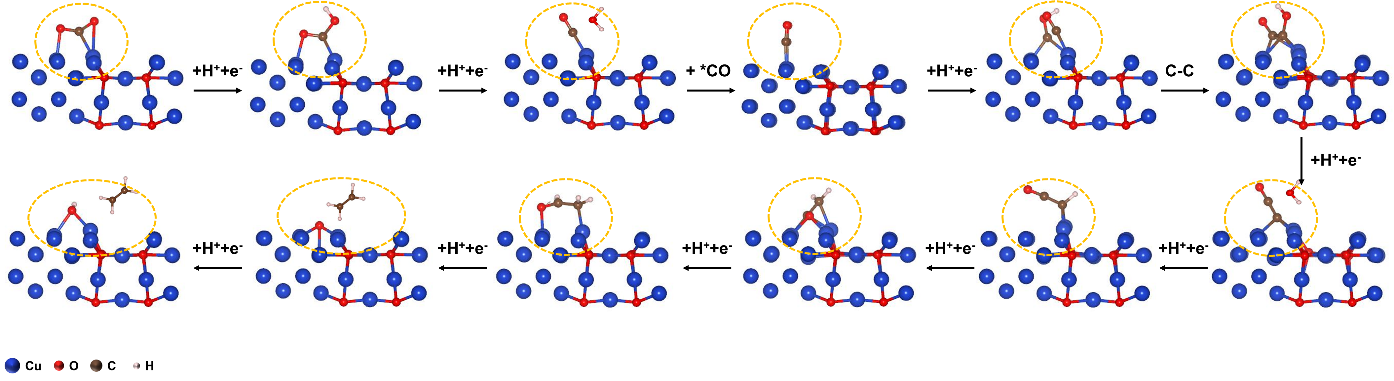


**Fig. S29 DFT simulation.** Optimized structures for the reaction intermediates of the C_2_H_4_ formation pathway on the Cu/Cu_2_O interface


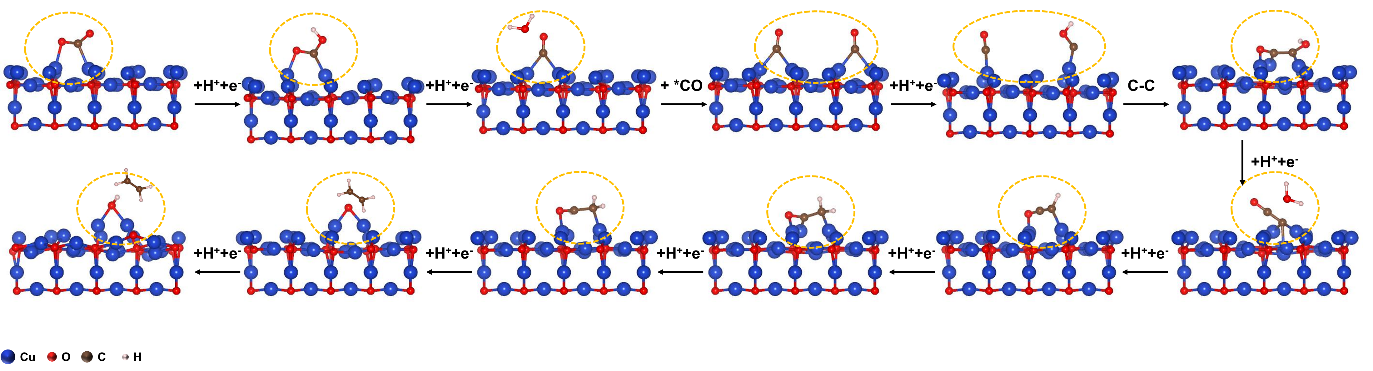


**Fig. S30 DFT simulation.** Optimized structures for the reaction intermediates of the C_2_H_4_ formation pathway on the Cu_2_O (110) slab


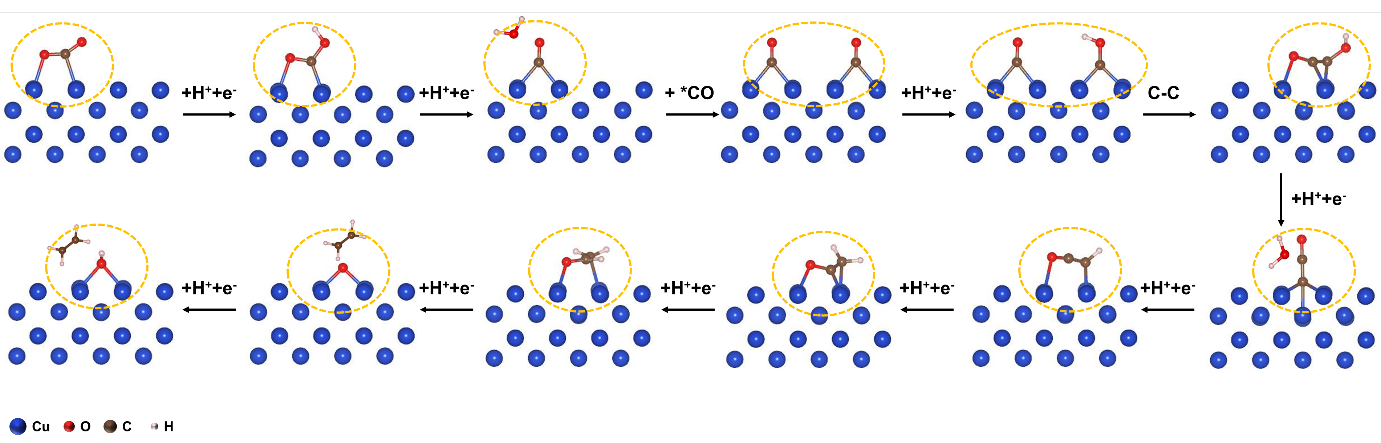


**Fig. S31 DFT simulation.** Optimized structures for the reaction intermediates of the C_2_H_4_ formation pathway on the Cu (110) slab


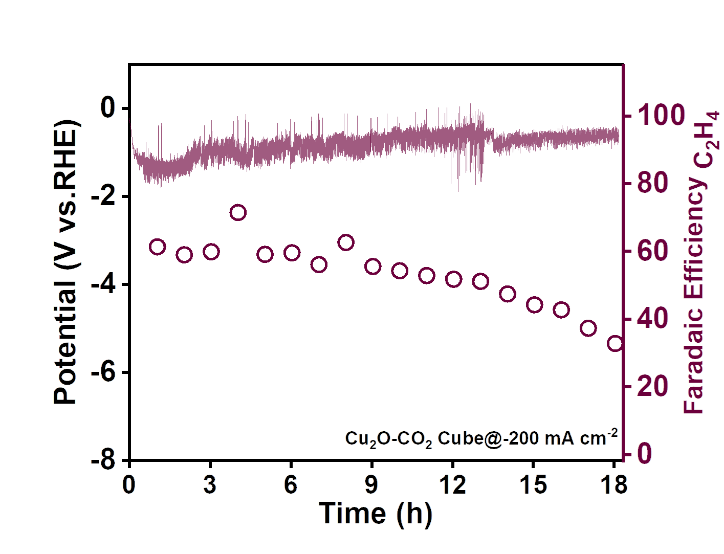


**Fig. S32** Stability of Cu_2_O-CO_2_ Cube at a current density of -200 mA cm^-2^ in a flow cell


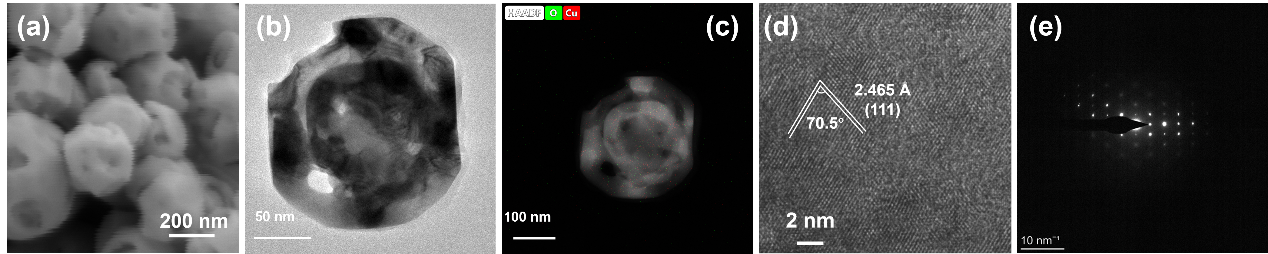


**Fig. S33 Microstructures of Cu_2_O hollow spheres.** **a**) SEM image, **b**) TEM image, **c**) EDS mapping, **d**) HRTEM, and **e**) SAED


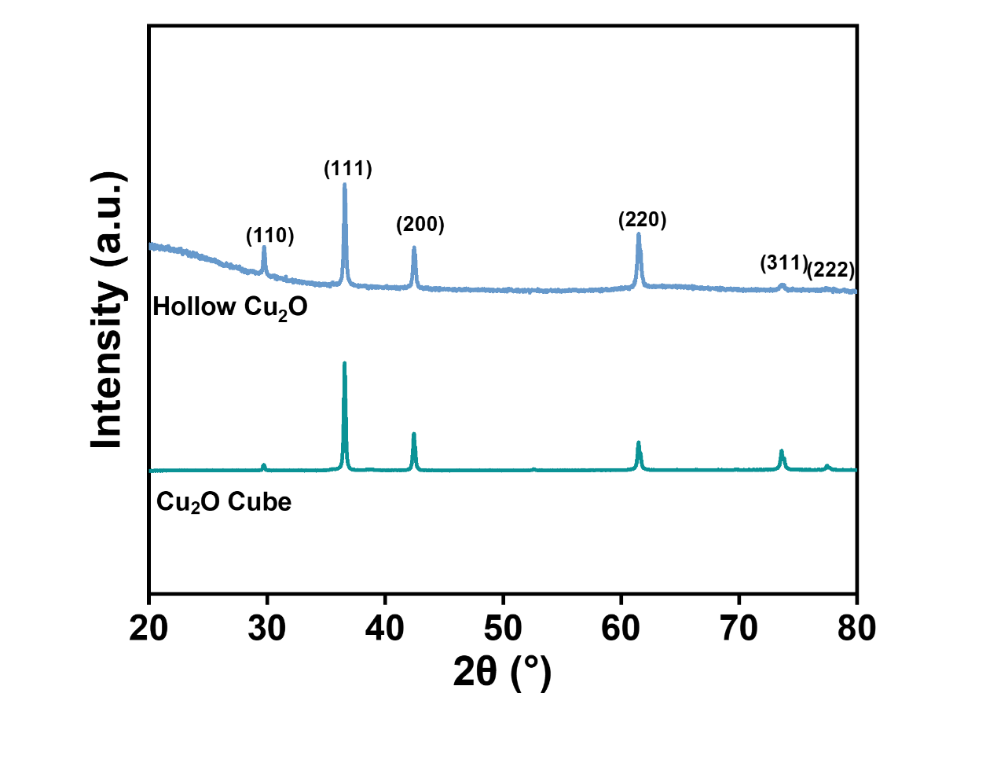


**Fig. S34** XRD patterns of Cu_2_O hollow spheres compared with Cu_2_O nanocrystals

**Fig. S35** Potential and Faradaic efficiency of hollow Cu_2_O-CO_2_, solid Cu_2_O-CO_2,_ and solid Cu_2_O-Ar at a current density of -200 mA cm^-2^


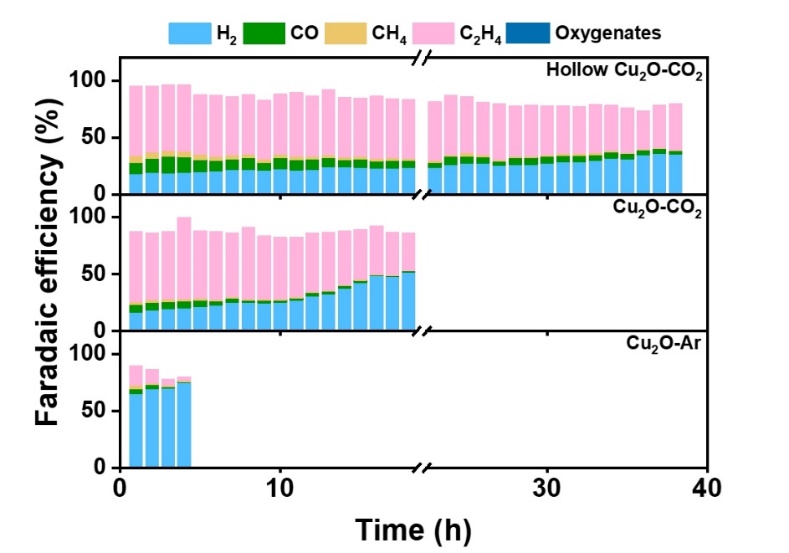


**Fig. S36** Faradaic efficiency of main CO_2_RR products after activation under flow cell configuration. For hollow Cu_2_O-CO_2_ and Cu_2_O-CO_2_, the data point with FE over 30% was shown. For Cu_2_O-CO_2_, the FE was much less than the threshold of 30%. Therefore, we only examined the sample for the initial 4 hours. It shows that hollow Cu_2_O-CO_2_ showed better stability


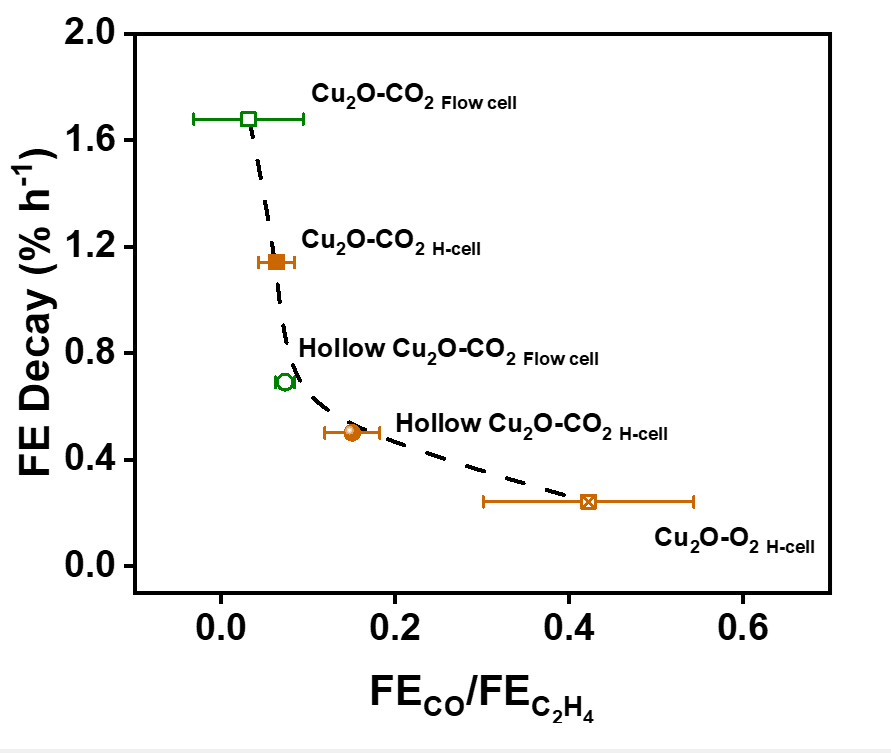


**Fig. S37** Correlations between FE_CO_/FE_C2H4_ and FE decay of all samples without Cu_2_O-Ar and Hollow Cu_2_O-Ar


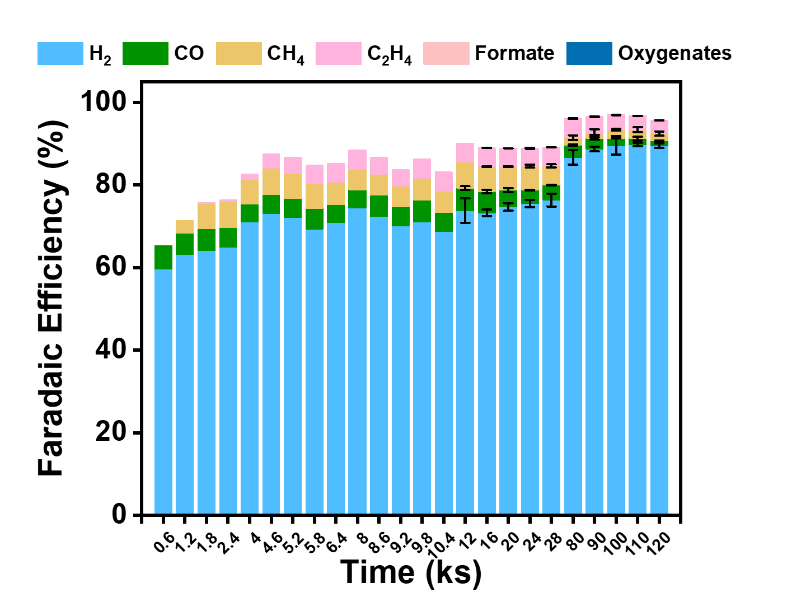


**Fig. S38** Faradaic efficiency of main CO_2_RR products after activation in Ar for Hollow Cu_2_O

**Table S1** Elemental distribution of pristine Cu_2_O nanocubes

| Z | Element | Family | Atomic fraction | Mass fraction | Fit error |
| --- | --- | --- | --- | --- | --- |
|  |  |  | % | % | % |
| 8 | O | K | 23.33 | 7.12 | 0.463556 |
| 29 | Cu | K | 76.67 | 92.88 | 0.212688 |

**Table S2** Elemental distribution of Cu_2_O-CO_2_

| Z | Element | Family | Atomic fraction | Mass fraction | Fit error |
| --- | --- | --- | --- | --- | --- |
|  |  |  | % | % | % |
| 8 | O | K | 27.39 | 8.68 | 2.38428 |
| 29 | Cu | K | 72.61 | 91.32 | 0.438095 |

**Table S3** Elemental distribution of Cu_2_O-Ar

| Z | Element | Family | Atomic fraction | Mass fraction | Fit error |
| --- | --- | --- | --- | --- | --- |
|  |  |  | % | % | % |
| 8 | O | K | 6.33 | 1.67 | 2.38428 |
| 29 | Cu | K | 93.67 | 98.33 | 0.438095 |

**Table S4** Comparisons of the catalytical performance towards C_2_H_4_ using Cu_2_O-based materials

| Catalyst | FEC_2_H_4_  (%) | C_2_H_4_ partial current density  (mA cm^-2^) | Applied potential  (V vs. RHE) | Stability  (h) | Cell type | Electrolyte | Refs. |  |
| --- | --- | --- | --- | --- | --- | --- | --- | --- |
| Cu-CuI | 71 | -276 | -0.87 | 85 | Flow cell | 1 M KOH | [S1] |  |
| ERD-Cu | 40 | -22 | -1.2 | 1 | H cell | 0.1 M KHCO_3_ | [S2] |  |
|  | 36 | -161 | -1.0 | 5 | Flow cell |  |  |  |
| Cu-CO_2_-60 | 60 | -174.5 | -3.7(E_full-cell_) | 68 | MEA | 0.15 M KHCO_3_ | [S3] |  |
| F-Cu_2_O | 74.1 | -14.69 | -1.2 | 12 | H cell | 0.1 M KHCO_3_ | [S4] |  |
| Cu_2_O(CO) | 55 | -275 | / | 6 | Flow cell | 1 M KOH | [S5] |  |
| Multihollow Cu_2_O | | 38 | -124.6 | -0.61 | 3 | Flow cell | 2 M KOH | [S6] |
| Cu_2_O-2 | 45 | -315 | -0.68 | 3 | Flow cell | 2 M KOH | [S7] |  |
| h-Cu_2_O ONSs | 43.5 | -8.92 | -1.1 | 4 | H cell | 0.1 M KHCO_3_ | [S8] |  |
|  | 42.8 | -85.6 | / | 4 | Flow cell | 1 M KOH |  |  |
| Cu_2_O-BN | 16 | -6.19 | -1.4 | 14 | H cell | 0.5 M KHCO_3_ | [S9] |  |
| CuAl-1 | 82.4 | -2.50 | -0.99 | 100 | H cell | 0.1 M KHCO_3_ | [S10] |  |
|  | 70.1 | -420.6 | -2.0 | / | Flow cell | 1 M KOH |  |  |
| t-Cu_2_O | 59 | -26.2 | -1.1 | 1.3 | H cell | 0.5 M KHCO_3_ | [S11] |  |
| Cu/CuSiO_3_ | 51.8 | -7.74 | -1.1 | 6 | H cell | 0.1 M KHCO_3_ | [S12] |  |
| Dual-phase Cu | 44.4 | -133.23 | / | 45 | Flow cell | 3 M KCl | [S13] |  |
| CuO-SH | 72 | -38 | -1.4 | 5 | H cell | 0.1 M KHCO_3_ | [S14] |  |
|  | 79.5 | -241.7 | -1.2 | 160 | Flow cell | 1 M KOH |  |  |
| C-NNu | 79 | -790 | -3.55(E_full-cell_) | 120 | MEA | 0.5 M KHCO_3_ | [S15] |  |
| **Hollow Cu_2_O** | 47 | -26.38 | -1.1 | 33 | H cell | 0.5 M KHCO_3_ | **Our work** |  |
|  | 61 | -122 | -0.6 | 48 | Flow cell | 1 M KOH |  |  |

**Supplementary References**

[S1] H. Li, T. Liu, P. Wei, L. Lin, D. Gao et al., High-Rate CO_2_ Electroreduction to C2+ Products over a Copper-Copper Iodide Catalyst, Angew. Chem. Int. Ed. **60**, 14329–14333 (2021). <https://doi.org/10.1002/anie.202102657>

[S2]P. De Luna, R. Quintero-Bermudez, C.-T. Dinh, M.B. Ross, O.S. Bushuyev et al., Catalyst electro-redeposition controls morphology and oxidation state for selective carbon dioxide reduction. Nat. Catal. **1**, 103–110 (2018). <https://doi.org/10.1038/s41929-017-0018-9>

[S3] Y. Wang, Z. Wang, C.-T. Dinh, J. Li, A. Ozden et al., Catalyst synthesis under CO_2_ electroreduction favours faceting and promotes renewable fuels electrosynthesis. Nat Catal **3**, 98–106 (2020). <https://doi.org/10.1038/s41929-019-0397-1>

[S4] H. Luo, B. Li, J.-G. Ma, P. Cheng, Surface modification of nano-Cu_2_O for controlling CO_2_ electrochemical reduction to ethylene and syngas. Angew. Chem. Int. Ed. **61**, e202116736 (2022). <https://doi.org/10.1002/anie.202116736>

[S5] Q. Wu, R. Du, P. Wang, G.I.N. Waterhouse, J. Li et al., Nanograin-boundary-abundant Cu_2_O-Cu nanocubes with high C2+ selectivity and good stability during electrochemical CO_2_ reduction at a current density of 500 mA/cm^2^. ACS Nano **17**, 12884–12894 (2023). <https://doi.org/10.1021/acsnano.3c04951>

[S6] P.-P. Yang, X.-L. Zhang, F.-Y. Gao, Y.-R. Zheng, Z.-Z. Niu et al., Protecting copper oxidation state via intermediate confinement for selective CO_2_ electroreduction to C2+ fuels. J. Am. Chem. Soc. **142**, 6400–6408 (2020). <https://doi.org/10.1021/jacs.0c01699>

[S7] X. Lv, Q. Liu, J. Wang, X. Wu, X. Li et al., Grain refining enables mixed Cu+/Cu0 states for CO_2_ electroreduction to C2+ products at high current density, Applied Catalysis B Environmental **324**, 122272 (2023). <https://doi.org/10.1016/j.apcatb.2022.122272>

[S8] M. Jun, C. Kwak, S.Y. Lee, J. Joo, J.M. Kim et al., Microfluidics-assisted synthesis of hierarchical Cu_2_O Nanocrystal as C2-Selective CO_2_ reduction electrocatalyst. Small Methods **6,**  2200074 (2022). https://doi.org/10.1002/smtd.202200074

[S9]Y. Zhou, Y. Yao, R. Zhao, X. Wang, Z. Fu et al., Stabilization of Cu^+^ *via* strong electronic interaction for selective and stable CO_2_ electroreduction. Angew. Chem. Int. Ed. **61**, e202205832 (2022). <https://doi.org/10.1002/anie.202205832>

[S10]S. Sultan, H. Lee, S. Park, M.M. Kim, A. Yoon et al., Interface rich CuO/Al_2_CuO_4_ surface for selective ethylene production from electrochemical CO_2_ conversion. Energy Environ. Sci. **15**, 2397–2409 (2022). <https://doi.org/10.1039/D1EE03861C>

[S11]Y. Gao, Q. Wu, X. Liang, Z. Wang, Z. Zheng et al., Cu_2_O Nanoparticles with both 100 and 111 facets for enhancing the selectivity and activity of CO_2_ electroreduction to ethylene. Adv. Sci. **7,** 1902820 (2020). <https://doi.org/10.1002/advs.201902820>

[S12]X. Yuan, S. Chen, D. Cheng, L. Li, W. Zhu et al., Controllable Cu^0^-Cu^+^ sites for electrocatalytic reduction of carbon dioxide. Angew. Chem. Int. Ed. **60,** 15344–15347 (2021). <https://doi.org/10.1002/anie.202105118>

[S13]P.-P. Yang, X.-L. Zhang, P. Liu, D.J. Kelly, Z.-Z. Niu et al., Highly enhanced chloride adsorption mediates efficient neutral CO_2_ electroreduction over a dual-phase copper catalyst. J. Am. Chem. Soc. **145**, 8714–8725 (2023). <https://doi.org/10.1021/jacs.3c02130>

[S14]Y. Yao, T. Shi, W. Chen, J. Wu, Y. Fan et al., A surface strategy boosting the ethylene selectivity for CO_2_ reduction and in situ mechanistic insights. Nat Commun **15**, 1257 (2024). <https://doi.org/10.1038/s41467-024-45704-2>

[S15]H. Wu, L. Huang, J. Timoshenko, K. Qi, W. Wang et al., Selective and energy-efficient electrosynthesis of ethylene from CO_2_ by tuning the valence of Cu catalysts through aryl diazonium functionalization. Nat. Energy volume **9**, 422–433 (2024). <https://doi.org/10.1038/s41560-024-01461-6>
